# Fluorescent modifications in aptamer switches—positional, structural, and neighboring pair effects on sensor performance

**DOI:** 10.1093/nar/gkaf1346

**Published:** 2025-12-12

**Authors:** Albert Zehan Li, Conrad H T Belzberg, Mimansa Sidhu, Amani A Hariri

**Affiliations:** Department of Chemistry, University of British Columbia, Vancouver, British Columbia V6T 1Z1, Canada; Department of Chemistry, University of British Columbia, Vancouver, British Columbia V6T 1Z1, Canada; Department of Chemistry, University of British Columbia, Vancouver, British Columbia V6T 1Z1, Canada; Department of Chemistry, University of British Columbia, Vancouver, British Columbia V6T 1Z1, Canada; Djavad Mowafaghian Centre for Brain Health, University of British Columbia, Vancouver, British Columbia V6T 1Z3, Canada

## Abstract

Aptamers are modular nucleic acid ligands increasingly used as recognition elements in biosensors for precision medicine. Their structure facilitates their adaptation into fluorescent switches through molecular design and conjugation with reporter molecules, which are typically selected based on optimal photophysical compatibility. However, a critical challenge persists: reporter molecules are not inert; their structure and underlying conjugation chemistry can introduce structural perturbations that adversely affect aptamer functional properties and DNA-duplex stability, subsequently impacting biosensing performance. Here, we evaluate the effect of fluorophore–quencher modifications by probing the interplay between reporter identity, local environment, and labeling positions within different switch architectures. We demonstrate that applying a combinatorial array of modifications to an aptamer switch sequence can tune its binding properties over a 2-order-of-magnitude range, with apparent binding affinities from ~230 to 2.3 μM and apparent kinetic response rates from ~1000 s to sub-second resolution, while also influencing the switch’s specificity and signal resolution. We also establish that the environment and spatial positioning of a reporter pair are critical design parameters, enabling order-of-magnitude performance differences even at theoretically suboptimal sites. This work provides a comprehensive study to help design optimal switches when using fluorescent modifications, enabling the development of next-generation biosensors with tailored performance.

## Graphical Abstract

**Graphical Abstract:**
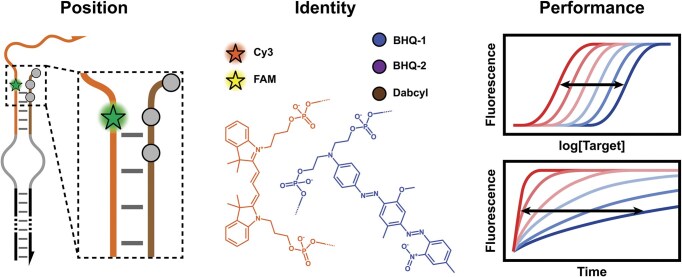

## Introduction

Aptamers, single-stranded nucleic acid molecules, represent a revolutionary class of recognition elements in biosensing and therapeutics [1]. Their fundamental sensing principle hinges on reporting the change in the target-dependent equilibrium of the conformational state of the aptamer (i.e. in the presence and absence of the target) achieved through different binding mechanisms [2]. This conformational change is the critical event that can be transduced into a measurable signal, thereby enabling their utility as versatile, reversible and robust biosensors (e.g. aptamer-switch) [3, 4]. Among the diverse signal transduction mechanisms, fluorescence resonance energy transfer (FRET) is a widely adopted strategy in aptamer switches, leveraging the proximity-dependent energy transfer between integrated donor and acceptor fluorophores and quenchers to report on these conformational dynamics [5, 6]. Dyes are strategically incorporated through various methods, including covalent attachment at the 5′ or 3′ termini, internal linkage to the phosphate backbone or a nucleobase, and non-covalent intercalation [7–9]. The selection of these fluorescent dyes and quenchers is primarily driven by their direct photophysical characteristics. Factors such as optimal FRET efficiency, which depends on spectral overlap, donor quantum yield, and dipole orientation (governed by the Förster distance, R_0_), are paramount [10, 11]. Similarly, the efficacy of dark quenchers, the minimization of background fluorescence for a high signal-to-noise ratio [12, 13], and robust photostability [14] for sensor longevity are critical considerations. Also, understanding a dye’s environmental sensitivity to parameters like the identity of neighboring nucleobases [15–17], pH, or temperature, is essential for accurate signal interpretation [18]. However, while these photophysical properties play a critical role in dye selection, the potential impact on the aptamer’s structure, conformation, and function, given the significant size, charge, and hydrophobicity of these modifications, is often dismissed.

Here, we demonstrate that the choice of fluorophore–quencher pair modifications, often perceived as passive reporters, can subtly or significantly alter the structure-switching capability of aptamer-based sensors. We investigate their impact on key sensor properties, including affinity, kinetics, specificity, and ultimately the sensor’s operating range. Our different combinations of modifications tested—all constructs are derived from a single switch architecture based on an established parent aptamer [19]—exhibit apparent affinities spanning two orders of magnitude, with equilibrium constants (*K*_d_) ranging from ~230 to 2.3 μM, and a kinetic response rate ranging from ~1000 s to sub-second resolution. A key strategy we identified to mitigate the apparent affinity cost associated with these modifications is the introduction of an internal offset between the reporter pair, which improved apparent affinity and kinetics by almost an order of magnitude. Lastly, we demonstrate that the local environment and position of a reporter modification are as critical to sensor performance as its photophysical properties. We investigated different labeling positions predicted to yield a suboptimal change in donor-acceptor distance upon target binding and found that the intrinsic properties and local interactions of the fluorophore–quencher pair at these positions can compensate for the affinity cost, generating a 100-fold better affinity and kinetics, at the expense, however, of specificity and operating range.

Remarkably, in this study, we show that the magnitude of this impact on affinity is not entirely correlated with duplex stability [20–25]. The effect is not generalizable; rather, it is highly dependent on the design, sequence, and target. Hence, the influence of these modifications on sensor performance is largely unpredictable based on current models. Our findings highlight that failing to account for these non-photophysical effects creates a significant barrier to optimal sensor design.

## Materials and methods

### DNA-based aptamer switch design

**Figure 1.**
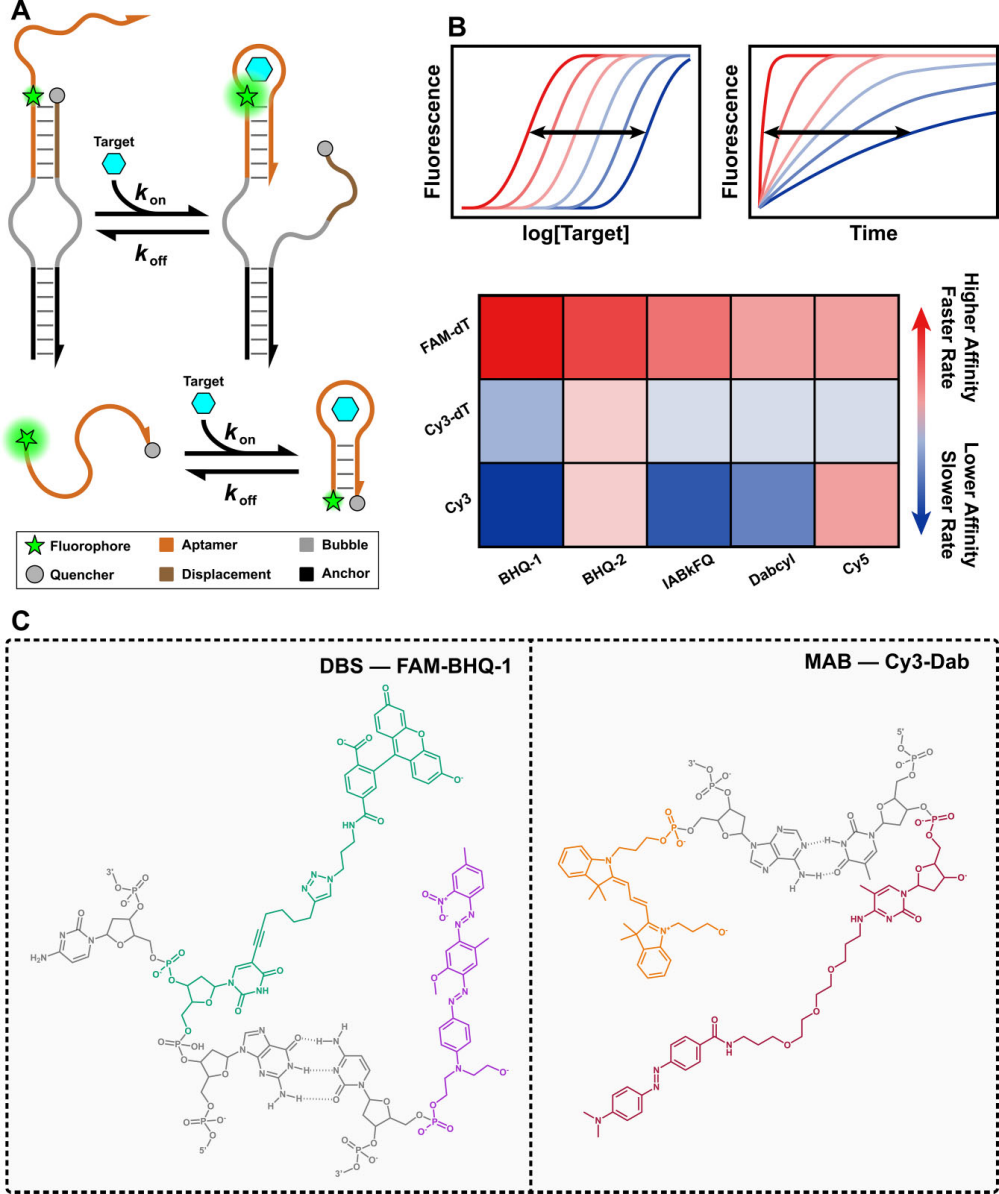
Evaluating the impact of fluorescent modifications on aptamer sensor performance. (**A**) Target-binding scheme of the switch designs employed. We chose the DBS (top) and MAB (bottom) designs to convert an existing aptamer into a switch, ON and OFF signal for the DBS and MAB, respectively. (**B**) We evaluate the influence of fluorophore–quencher pair identity and position on the apparent binding affinity and kinetics of aptamer-based switches. (**C**) Example structures of fluorescent modifications used and their different bioconjugation strategy depending on their identity and position. Left: 6-FAM incorporated internally on a thymidine by click chemistry adjacent to 5′ BHQ-1, in the DBS. Right: 5′ Cy3 adjacent to 3′ Dab incorporated on a 5-methyldeoxycytidine, in the MAB. Structures of all modifications used in this work are illustrated in Supplementary Fig. S1.

We examined how the identity and position of a fluorophore–quencher pair influence the key performance metrics: apparent binding affinity and kinetics of aptamer-based switches. For this purpose, we employed the well-established duplex-bubble switch (DBS) and molecular aptamer beacon (MAB) as model systems, which were modified with fluorescent pairs (Fig. 1A and B) [26, 27].

Their actuation works as follows: Capture-SELEX aptamers with a hairpin architecture comprise a random nucleotide region, evolved during the selection for target binding, flanked on both sides by a constant stem [28, 29]. The principle behind these aptamer-based biosensors relies on exploiting a significant conformational rearrangement (characterized by a target-sensor-based equilibrium) that serves as the core of the signal transduction mechanism [2]. Often, this structural rearrangement and consequent signal change are not significant or nonexistent [30, 31]. To circumvent this issue, aptamers can be coupled to a strand displacement assay [32, 33] and adapted to a DBS architecture, or truncated as in the MAB (Fig. 1A). In such systems, a structural difference between the bound and unbound states alters the distance between the fluorescent pair, resulting in photophysical effects via direct contact (static or collisional quenching) or energy transfer through FRET. Due to this proximity dependence and the design of these aptamer switches, the fluorescent modifications are likely to be positioned in closer proximity either in target-unbound (DBS) or -bound states (MAB), allowing for potential direct contact. The DBS, an ON switch, transitions from an initial state where a modification pair is closely positioned (signal off/low) to a target-bound state where the pair separates (signal on/high) (Fig. 1A, top). The MAB, an OFF switch, operates in reverse: its fluorescent labels are initially distanced (signal on/high in some FRET contexts, or simply unquenched), and target binding promotes their convergence, leading to a signal change (e.g. FRET or static quenching, signal off/low) (Fig. 1A, bottom).

### Fluorophore/quencher choice and attachment strategy

The fluorophores and quenchers used in this study were chosen based on their widespread use and economic considerations. Specifically, cyanine dyes, and fluorescein to a lesser extent, have been widely used since they are available as phosphoramidites and are easy to attach to nucleic acids. Cyanine3 (Cy3) and 6-carboxyfluorescein (6-FAM) were selected as donor fluorophores (Fig. 1C and Supplementary Fig. S1). The acceptor quenchers included dabcyl (Dab), Black Hole Quenchers 1 and 2 (BHQ-1/2), and Iowa Black FQ (IABkFQ). Dabcyl functions predominantly as a static quencher, while Black Hole Quenchers 1 and 2, and Iowa Black FQ utilize more collisional- and FRET-quenching interactions (Fig. 1C and Supplementary Fig. S1) [22, 34]. We characterized ligand binding across an array of aptamer switches, all based on the same dopamine aptamer sequence (Supplementary Tables S1 and S2). To explore the impact of labeling, we systematically varied the fluorophore–quencher pairs, their attachment positions (e.g. internal linkage to the phosphate backbone or a nucleobase), and the local DNA steric environment (e.g. double-stranded regions versus more flexible single-stranded regions). Modifications were positioned outside of the hairpin loop, which has been previously identified as part of the binding domain [27, 35]; however, we acknowledge the possibility of more global structural consequences of bioconjugation. Ligand binding and sensor response rate were then assessed by monitoring fluorescence changes as a function of target concentration or time, respectively, using a plate reader-based assay.

### Chemicals and materials

All DNA were synthesized by Integrated DNA Technologies (Coralville, IA, USA) and their corresponding sequences are reported in Supplementary Tables S1 and S2, or in figure captions. Dopamine hydrochloride (H8502), norepinephrine bitartrate salt (N-5785), D-(+)-glucose (G7528), and hydrocortisone (H0888) were purchased from Sigma–Aldrich. The 10X PBS buffer (AM9624), 1 M Tris–HCl (pH 7.5) (15567-027), magnesium chloride (AM9530G), reagent alcohol (A962), serotonin hydrochloride (B21263.03), and adenosine 5′-triphosphate sodium salt (R0441) were purchased from Thermo Fisher Scientific. The 0.2 mL 8-tube PCR strips without caps, low profile white (TLS0851), and optical flat 8-cap strips for 0.2 mL tube strips/plates (TCS0803) were purchased from Bio-Rad Laboratories. The 96-well half-area, flat-bottom, non-treated, black polystyrene plates (3694) were purchased from Corning Incorporated. HyPure™ Molecular Biology Grade Water (SH30538.03) was purchased from Cytiva and used for the preparation of the annealing buffer.

### Binding curves for the determination of the apparent equilibrium dissociation constant

All DNA constructs with various fluorescent modifications and labeling positions were resuspended to a final concentration of 200 × 10^−9^ M in binding buffer A [1× PBS (phosphate-buffered saline), pH 8.0, and 2 × 10^−3^ M MgCl_2_], with the exception of the ATP intramolecular strand displacement (ISD) switches, which were resuspended to a final concentration of 250 × 10^−9^ M in binding buffer B (10 × 10^−3^ M Tris–HCl, pH 7.5, and 6 × 10^−3^ M MgCl_2_). DBS constructs were prepared by mixing 1 × 10^−6^ M aptamer strand with 4 × 10^−6^ M displacement strand at a 1:4 ratio, unless otherwise specified. MAB, ATP ISD, and serotonin switches were initially diluted to 1 × 10^−6^ M before annealing. DBS and MAB constructs were annealed at 95°C for 2 min, then cooled to room temperature over 30 min to achieve maximal hybridization. ATP ISD and serotonin constructs were annealed at the same temperature, except for 5 min. To obtain binding curves in solution, 40 µl reactions were prepared in the appropriate binding buffer with DNA construct and final target concentrations (corresponding to the aptamer employed) in the range of 0 to 2 × 10^−3^ M for dopamine DBS and MAB, 0 to 2 × 10^−6^ M for serotonin DBS, 0 to 2 × 10^−5^ M for the serotonin sensor, and 0 to 4 × 10^−3^ M for the ATP ISD switch. Stock solutions of 100 × 10^−3^ M for all targets were prepared in the appropriate binding buffer, with the exception of hydrocortisone, which was initially diluted to 30 × 10^−3^ M in reagent alcohol (88%–91% ethanol), and ATP solution, which was purchased at 100 × 10^−3^ M in water. The fluorescence spectra for all samples were measured at 25°C on a Tecan Spark multimode microplate reader (Supplementary Fig. S2). Prior to spectral analysis, sensors were incubated with their target molecules. Incubation times were optimized for each sensor type: 5 min for dopamine DNA-based sensors (DBS) and MABs to prevent polydopamine formation (Supplementary Figs S3 and S4, and Supplementary Table S3), 30 min for serotonin DBS, 40 min for most serotonin sensors (70 min for the Cy3–Cy5 sensor), and 15 min for the ATP ISD switch. Fluorescence emission spectra for Cy3-labeled sensors were acquired from 560 to 700 nm using an excitation wavelength of 520 nm. Specific constructs required a modified excitation of 515 nm, including Cy3-BHQ-1/BHQ-2 MABs and Cy3-BHQ-1/Dab ATP ISD switch. The detector gain was set to 100 for most measurements, but was increased to 130 for Cy3-BHQ-1/BHQ-2 MABs and 120 for Cy3-BHQ-1/Dab ATP ISD constructs to optimize signal detection. For Cy5, emission spectra were monitored in 660–800 nm range with excitation at 620 nm and a gain of 100. For FAM, emission spectra were monitored in the 515–650 nm range with excitation at 475 nm and a gain of 90 for the DBS and the FAM–TAMRA serotonin sensor, and 100 for the MAB and ATP ISD switches. All *K*_d_ determinations were from binding curves plotted at the maximum fluorescence emission. Measurements were conducted in a Corning 96-well half-area black flat-bottom polystyrene microplate. Representative concentration-dependent emission spectra are shown in Supplementary Fig. S2. Binding curve fits are described in Supplementary Note 1. Briefly, the apparent equilibrium dissociation constant (*K*_d_) was obtained by fitting the binding curves (background corrected and normalized) to the one-site binding model (Hill1, where *n* = 1) using the Origin software. For more accurate fitting, concentrations that were significantly impacted by polydopamine formation were excluded.

### Kinetic traces for the determination of the time constant (*τ*)

DNA constructs were prepared as described above. Initially, DNA constructs with various fluorescent modifications and labeling positions were suspended at a concentration of 240 × 10^−9^ M in 30 µl binding buffer. Kinetic fluorescent measurements were achieved using a Tecan Spark multimode microplate reader. After timed injection of 10 µl of 100 × 10^−6^ M dopamine in binding buffer into the 30 µl DNA solution to achieve a final concentration of 25 × 10^−6^ M dopamine and 200 × 10^−9^ M DNA construct (40 µl), the kinetic response was measured using monochromators at a regular time interval between 1–15 s, in which the accuracy of determining the time constant was not compromised by the frequency of the read. First, a stable baseline was established by allowing the DNA solution to equilibrate until its fluorescence intensity changed by <5% per minute. Following the addition of the target, the fluorescence was monitored until the signal reached a new equilibrium, as defined by the same criterion. Measurement parameters were the same as described above (except for the Cy3–BHQ-2 MAB, which was measured at a gain of 132). Kinetic data were not corrected relative to the target free control to account for the effect of sample volume change upon injection of dopamine as in many cases there was an overcorrection. For plotting, the curves were normalized to a range of 0–1 in order to visually emphasize changes in rate constants rather than the background and peak levels that were dictated by the thermodynamics. These data were normalized as described in Supplementary Note 1. Each replicate was normalized, and the normalized datasets were then averaged. Kinetic trace fits are described in Supplementary Note 1. Briefly, the observed time constant (*τ*) was obtained by fitting the data to a one-phase association curve in Origin (Supplementary Fig. S5).

### DNA melting curves for the determination of the melting temperature (*T*_m_)

DNA constructs were prepared as described above. Initially, DNA constructs with various fluorescent modifications and labeling positions were suspended at a concentration of 200 × 10^−9^ M in 20 µl binding buffer, in the absence and presence of 50 µM dopamine. Fluorescence-based melting curve traces, in triplicate, were achieved using the CFX Opus 96 Real-Time PCR System. Samples were incubated at 25°C for 3 min, then the temperature was ramped up by 0.2°C (rate of temperature change: 0.1°C/s), followed by a 30 s hold at each 0.2°C increment, until a final temperature of 95°C was reached (Supplementary Fig. S6). Software-automated melting curves had their background subtraction removed using the CFX Maestro Software. Raw data were then processed using Origin. Raw melting curves for the DBS and MAB were corrected for the fluorophore temperature-dependent decrease by normalizing to the melting curve trace of the isolated fluorophore (Cy3 or FAM)-labeled aptamer DBS strand in the absence or presence of 50 µM dopamine (i.e. the melting curve of an FAM–DBS construct in the presence of 50 µM dopamine was normalized to the melting curve of FAM-labeled DBS aptamer strand in the presence of 50 µM dopamine). To calculate the melting temperature (*T*_m_), the first derivative plot of the corrected melting curves was computed with smoothing using the Savitsky–Golay filter (polynomial order 2 and 20 points of window) and the *T*_m_ was determined to be the temperature associated with the maximum first derivative value (or an average of two temperatures in the case of equal first derivative values).

## Results

### Interplay of fluorophore/quencher properties and labeling position on sensor function

#### Impact of identity and local environment at a photophysically optimized labeling site

**Figure 2.**
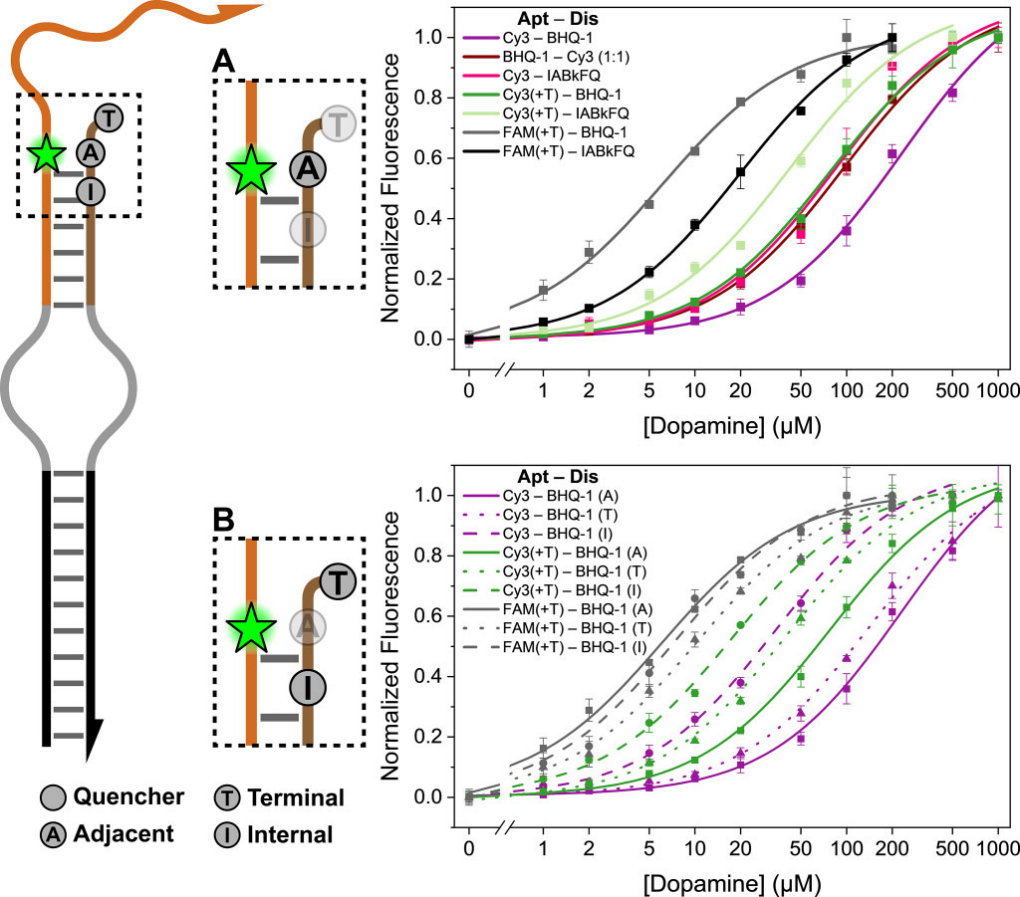
Impact of fluorescent modification identity and offset position on DBS switch apparent binding affinity at a photophysically optimized labeling site. An array of DBS aptamer-based switches with varying pairs of fluorescent modifications positioned at either the (**A**) adjacent position (A) or (**B**) terminal (T) or internal (I) offset positions are evaluated by their apparent affinities. Structures of fluorescent modifications at their respective positions are provided in Supplementary Fig. S1. (A) At the adjacent position A, swapping a fluorophore from Cy3 to FAM causes a large binding curve shift (2 orders of magnitude change in *K*_d_). (B) Introducing an offset between the fluorophore (Cy3) and the adjacent quencher (BHQ-1) recovers the system’s apparent binding affinity up to an order of magnitude. All plots are averaged over three replicates (*n* = 3). Error bars in panels (A) and (B) represent the standard deviation of the average. Data normalization and fits are detailed in the “Materials and methods” section and in Supplementary Note 1. Sample raw thermodynamic plots, heatmaps, and relevant parameters for the constructs are provided in Supplementary Figs S2 and S7, and Supplementary Tables S4 and S5. Source data for all binding curves are provided as a “Source data” file.

To understand the role of dye properties (chemical structure, charge, hydrophobicity) in target recognition and binding kinetics, we systematically investigated how altering fluorophore–quencher identity impacts the apparent affinity of our aptamer in a DBS system (Fig. 2). For our initial experiments, we focused on a specific fluorophore–quencher labeling position within the DBS architecture (Fig. 2A, Supplementary Figs S2 and S7, and Supplementary Tables S4 and S5). The selection of this position was guided by a theoretical model of the sensor’s conformational change. This model assumes an ideal, best-case scenario in which the complementary strand is completely displaced in a target-bound state, which is predicted to yield the maximal change in donor–acceptor distance (i.e. aptamer–displacement separation) (Supplementary Note 2) [26]. Such a strategy, prioritizing the largest possible distance change, is typical in FRET-based sensor design, where the primary consideration is often optimizing photophysical parameters like FRET efficiency to achieve the best theoretical signal enhancement and signal-to-background ratio.

Using this photophysically optimal labeling site, we first observed that substituting the fluorophore FAM for Cy3 (while keeping the quencher BHQ-1 constant) significantly decreased the apparent affinity (Fig. 2A). This change was substantial, raising the *K*_d_ from 6.1 ± 0.7 μM (FAM—BHQ-1) to 229.9 ± 22.0 μM (Cy3–BHQ-1)—a nearly 50-fold difference as observed in Fig. 2A. The observed reduction in apparent *K*_d_ with FAM can be partly attributable to its incorporation method; internal coupling to thymine likely destabilizes the aptamer–displacement duplex (Supplementary Fig. S8). To isolate this specific effect, we evaluated Cy3 (conjugated within the phosphate backbone) on an equivalent thymine-supplemented sequence [Cy3(+T)–BHQ-1], which yielded an intermediate *K*_d_ of 76.7 ± 5.5 μM (Fig. 2A). This suggests that the thymidine incorporation itself contributes ~1 order of magnitude to the overall affinity improvement when comparing Cy3–BHQ-1 to FAM–BHQ-1. This result prompts a hypothesis regarding the dianionic nature of FAM, which could introduce electrostatic interactions (e.g. anionic repulsion with the DNA backbone or nucleobases) not present with Cy3 [17, 36]. Furthermore, existing research on Cy3’s interactions with nucleic acids suggests its behavior can be complex and context-dependent, potentially involving specific stacking preferences or altered duplex stability that might not be fully captured by simple steric or sequestration models [37, 38].

To investigate whether the local environment and steric factors surrounding the Cy3 label, when incorporated into the DNA backbone, play any significant role in influencing affinity and kinetics, we reversed the strand assignments, comparing our standard internally labeled Cy3–BHQ-1 construct to one where Cy3 was placed terminally (BHQ-1–Cy3). It is important to note that while our typical DBS system employs a 1:4 aptamer-to-displacement strand ratio (i.e. simulations predict the formation of higher-order structures that we speculate impede aptamer binding capability as illustrated in Supplementary Fig. S9), this specific comparison necessitated a 1:1 ratio for optimal background signal and thus reliable data fitting. At this 1:1 ratio, we observed that placing Cy3 terminally resulted in a significant two-fold increase in affinity (from Cy3–BHQ-1 at a 1:1 ratio), with the *K*_d_ decreasing to 95.6 ± 6.7 μM (Supplementary Fig. S9 and  Supplementary Table S6). This finding suggests that Cy3’s specific environment along the backbone does have an important influence on the apparent affinity. However, despite this improvement, the *K*_d_ remains ~15-fold greater than that observed with FAM, indicating that steric effects from neighboring bases, while influential, are not the sole factor driving the overall difference in affinity.

While we have established that Cy3’s environment can influence affinity, it does not fully account for the substantial difference compared to FAM. This led us to further investigate the hypothesis that specific structural interactions between the Cy3 fluorophore and the BHQ-1 quencher are responsible for the observed decrease in apparent affinity, rather than just steric hindrance, electrostatic character, or other general effects. Since an increase in effective *K*_d_ indicates greater difficulty in displacing the competing DNA strand, the substantially higher *K*_d_ of Cy3–BHQ-1 compared to FAM–BHQ-1 strongly implies that Cy3 has a more significant structural impact on the aptamer. This is likely due to enhanced interactions (i.e. electrostatics) with its immediate chemical environment, including adjacent bases, backbone, and/or modifications. Excluding electrostatic-mediated dye–DNA interactions, one other possibility is a specific structural interaction between the Cy3 and the BHQ-1 modifications that increases the hybridization strength of the DBS system, shifting the *K*_d_ upward. To validate that this reduced affinity is indeed caused by such specific physical interactions, we paired Cy3 with IABkFQ instead, a quencher with a distinctly different structure and thus a different presumed mode of interaction with Cy3. This substitution resulted in a *K*_d_ of 85.0 ± 15.6 μM, which is two-fold lower than Cy3–BHQ-1, consistent with the idea that the Cy3 and/or BHQ-1 can favorably form stabilizing interactions with their surrounding chemical environment—specific Cy3–BHQ-1 dye–dye interactions or dye–DNA interactions.

In order to lessen such structural interactions caused by the modifications themselves, without significantly altering the distance change critical for FRET or FRET efficiency, our strategy was to “offset” previously adjacent fluorescent modifications (Fig. 2B). We selected the most affinity-impacted combination, Cy3–BHQ-1, and investigated the effect of shifting BHQ-1 by one nucleotide, either by moving the modification internally or by the terminal addition of a 5′ thymidine (Fig. 2B). We observed that terminally offsetting the quencher improved apparent binding affinity approximately two-fold by decreasing the *K*_d_ from 229.9 ± 22.0 to 142.9 ± 11.7 μM, while an internal modification improved affinity by about seven-fold to 34.0 ± 3.3 μM. These trends were sustained using Cy3(+T) and BHQ-1, where the internal offset proved to be superior to that of the terminal, presumably because the terminal offset permits sufficient flexibility that allows the quencher to interact with Cy3 or with the DNA backbone, nullifying the distance displacement, whereas internally offsetting allows the quencher to be potentially sandwiched between the neighboring bases, weakening the displacement duplex and/or sequestering it from a fluorophore–quencher or reporter–DNA interactions, thereby increasing the effective affinity. Terminal offsets are therefore not ideal, as presumably increased distance from the partner modification is necessary to improve affinity, which is, however, limited by quenching efficiency and consequently sensor sensitivity and operating range. Finally, we tested the offsets with FAM(+T) and did not observe any significant difference in affinity to the standard adjacent positioning; 10.2 ± 1.0 μM terminally and 7.5 ± 0.9 μM internally. This finding further supports that the dianionic nature of FAM minimizes target binding impeding interactions, while the positive charge of Cy3 can lead to significant, consequential interactions.

**Figure 3.**
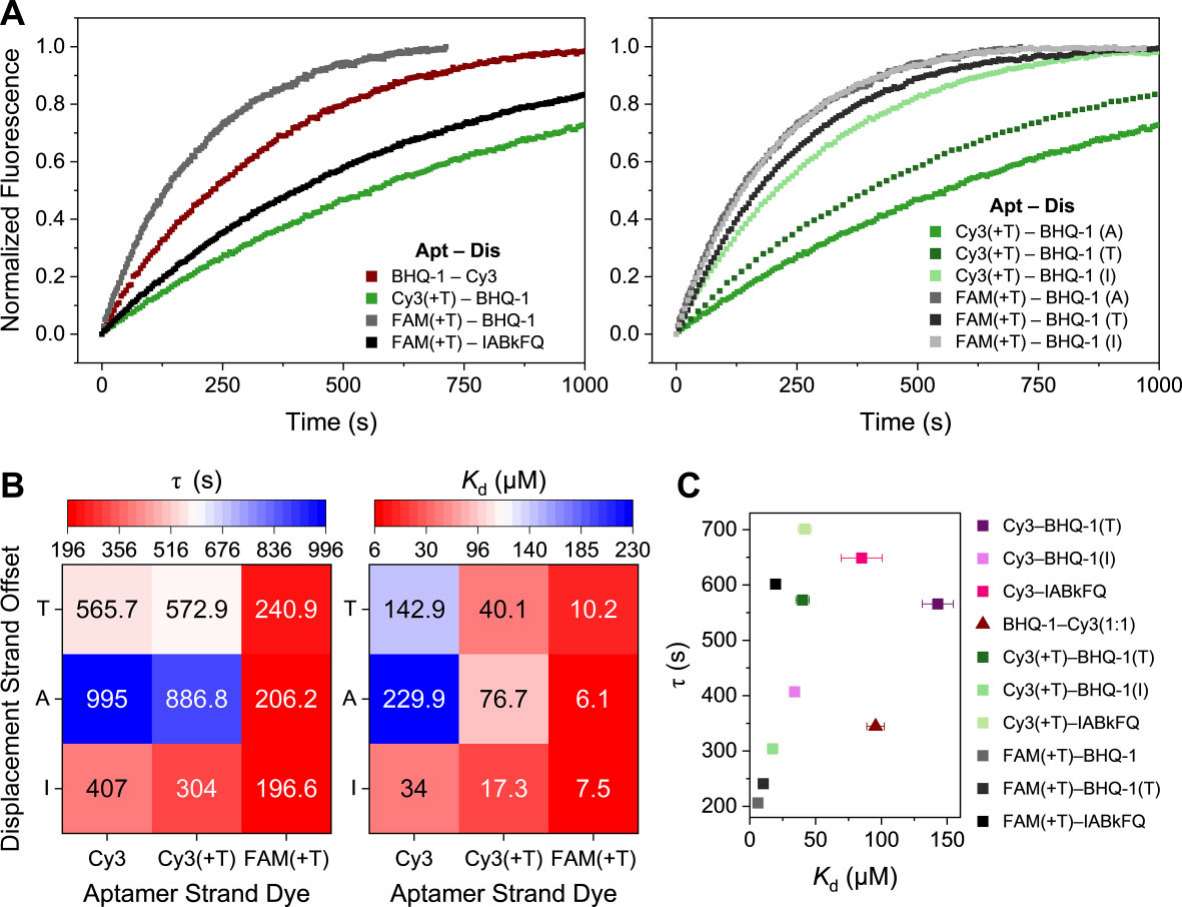
Impact of fluorescent modifications on temporal response. (**A**) Normalized signal change over time upon injection with 25 μM dopamine. While holding the position constant at the adjacent position(A), changing the identity of the modification resulted in a different kinetic response (left). While holding modification identity constant, shifting the position of the modification through a terminal (T) or internal (I) offset resulted in faster kinetics (right). (**B**) Kinetic (left) and thermodynamic (right) landscape of aptamer switches, illustrating the impact of varying the offset position of the Cy3 dye relative to the quencher. (**C**) Correlation plot illustrating the comparison of temporal response to apparent binding affinity for constructs with varied fluorophore–quencher combinations and offset positions. The data reveal that specific combinations can elicit vastly different kinetic responses even when the overall apparent binding affinity remains constant. Data normalization and fits are explained in the “Materials and methods” section and in Supplementary Note 1. Plots were averaged over three replicates (*n* = 3). Error bars in panel (A) were omitted for clarity and panel (C) represent the standard error. Standard errors for equilibrium dissociation and time constants in panels (A), (B), and (C) are listed in Supplementary Tables S4 and S5. Source data for all kinetic traces are provided as a “Source data” file.

Next, we sought to determine whether the modifications that impacted binding strength also influenced the response rate, to provide a better picture of their functional performance (Fig. 3). Upon injecting dopamine at a known concentration (25 μM), we monitored the fluorescence response over time to assess the kinetics of molecular recognition for each construct. We observed that the identity of the modification significantly influenced the temporal response. When positioned adjacently, the response time varied by up to four-fold, ranging from 200 s for FAM(+T)–BHQ-1 to 870 s for Cy3(+T)–BHQ-1 (Fig. 3A, left). Furthermore, offsetting the modification pair resulted in faster responses compared to the adjacent position. Specifically, internal offsets were faster than terminal offsets for all modifications except FAM(+T)–BHQ-1, for which no significant difference was observed, again highlighting the minimal impact of FAM-labeled constructs on sensor performance (Fig. 3A, right). Although a general correlation between affinity and kinetics was observed (better affinity, faster response), several exceptions were noted. For instance, Cy3 and Cy3(+T) at the terminal position, as well as Cy3(+T) at the adjacent position, exhibited poorer correlation (Fig. 3B). For some combinations, the influence of modifications on response rate proved to be more complex, exhibiting case-by-case significant differences when comparing all *K*_d_ and time constants relative to Cy3–BHQ-1. Modification pairs with similar affinities can have vastly different kinetics, and conversely, pairs with similar kinetics can exhibit markedly different affinities (Fig. 3C and Supplementary Fig. S10). This indicates that while modifications impact response rates, their influence is not always directly proportional to the change in affinity, suggesting complex underlying kinetic mechanisms and the potential to tune a desired apparent affinity and temporal response solely via the strategic selection of chemical modifications.

#### Beyond ideal FRET conditions: performance at alternative labeling positions

**Figure 4.**
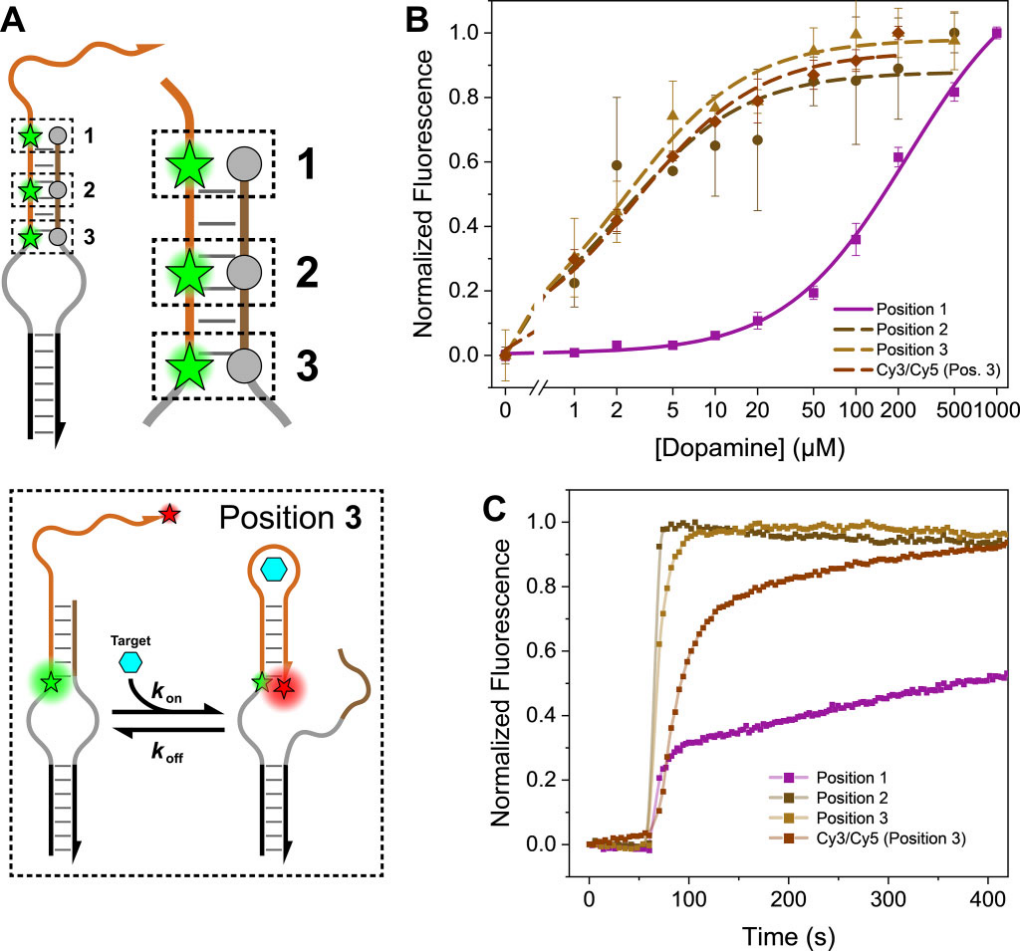
Impact of fluorescent modifications at suboptimal photophysical positions on aptamer affinity and kinetic response. (**A**) Modifications were introduced at two sites: the rigid stem (position 2) and the flexible loop (position 3), and are compared to the previously adjacent position (position 1). Inset: A FRET-based Cy3–Cy5 design at position 3 designed to enhance signaling performance. In this system, initially, in the target-unbound state, the construct predominantly exists in a state where Cy3 and Cy5 are distanced from each other with an enhanced Cy3 emission. After the introduction of the target, the equilibrium shifts to a state where Cy3 and Cy5 are adjacent to each other, and Cy3 emission diminishes. (**B**) Binding curves for different fluorescent modification positions. (**C**) Normalized signal change over time upon injection with 25 μM dopamine. Shifting the position of the modification pair from position 1 to positions 2 and 3 both resulted in a 100-fold improvement in apparent binding affinity and accelerated kinetics. Data normalization and fits are explained in the “Materials and methods” section and in Supplementary Note 1. Plots were averaged over three replicates (*n* = 3). Error bars in a are standard deviation and were omitted in panel (B) for clarity. Standard errors for equilibrium dissociation and time constants derived in panels (B) and (C), respectively, are listed in Supplementary Table S7.

Having established that the local environment, mobility, and interactions of fluorescent modifications significantly affect binding strength and response time, we next investigated how relocating the dye–quencher pair to a less FRET-optimal position impacts these metrics (Fig. 4 and Supplementary Table S7). Our aim was to determine whether altering the dyes’ environment (flexibility) and interactions by changing their position—even at the cost of ideal FRET parameters—would further influence affinity and kinetics. We therefore tested the modification pair, Cy3–BHQ-1, either both inserted in a rigid double-stranded environment (position 2) or a flexible single-stranded environment (position 3) (Fig. 4A), where they possessed greater degrees of freedom. These configurations were compared against the adjacent position (position 1), in which one modification was immobilized on the phosphate backbone and the second was attached to the 5′ terminus. We observed that for both of these environments, surprisingly, the apparent binding affinity improved by 100-fold (*K*_d_ = 2.2 ± 0.7 and 2.3 ± 0.3 μM for positions 2 and 3, respectively) (Fig. 4B). We also observed a significant improvement in the temporal response, which was enhanced almost 100-fold to 12 s in position 3. The kinetics for position 2 were even faster, exceeding the resolution of our measurement and preventing accurate fitting (Fig. 4C). This indicates that there is a high spatial probability that modifications positioned within a flexible spacer or a rigid duplex will be sequestered from participating in target-binding impeding interactions (i.e. stabilizing dye–dye, dye–duplex DNA interactions, and/or destabilizing dye–aptamer interactions). Recovering effective affinity by placing modifications within a different environment, even with a suboptimal FRET distance, is not without drawbacks for overall sensor performance. This apparent benefit is offset by increased background fluorescence and markedly poorer operating range (Supplementary Note 3 and Supplementary Table S8). Plausible explanations include an increased average distance between the fluorophore and quencher when tethered at these positions and a more limited range of motion (Supplementary Note 2). When comparing the operating range across all constructs, it is clear that dye flexibility has the potential to impact the relative orientation of the modifications, which can alter FRET efficiency and potentially contact quenching mechanisms known to be significant for BHQ-1.

Our results indicate that placing the fluorophore–quencher pair at position 2 and 3 mitigates the impact of the local chemical environment on aptamer performance but comes at the cost of a reduced operating range (Supplementary Table S8). This insight informs a new design strategy to overcome dye–dye interactions. We employed a FRET-based system that keeps the primary dye (Cy3) at the environmentally stable position 3 while introducing a FRET acceptor, such as Cy5, at the opposite end of the aptamer (Fig. 4A, inset). In this system, the DBS was modified to function as a signal-off switch through the quenching of Cy3 by FRET to Cy5 upon binding, ensuring no direct acceptor–donor interactions initially occur in the target-unbound state. The measured *K*_d_ of 9.9 ± 3.6 μM (Fig. 4B and C, Supplementary Fig. S11, and Supplementary Table S7) is similar to that of positions 2 and 3 and, notably, is comparable to the affinity of the FAM–BHQ-1 construct at position 1, which has presumed minimal dye interactions (Fig. 2A). Therefore, this newly designed FRET-switch maintains favorable apparent binding affinity and kinetics and can be a more robust and generalizable platform for the future (especially for applications that use cyanine dyes), achieving a minimal impact on the core binding properties by reducing nonspecific interactions between the fluorescent modifications and the DNA backbone.

### Generalizability of dye modification effects across different aptamer switch designs

**Figure 5.**
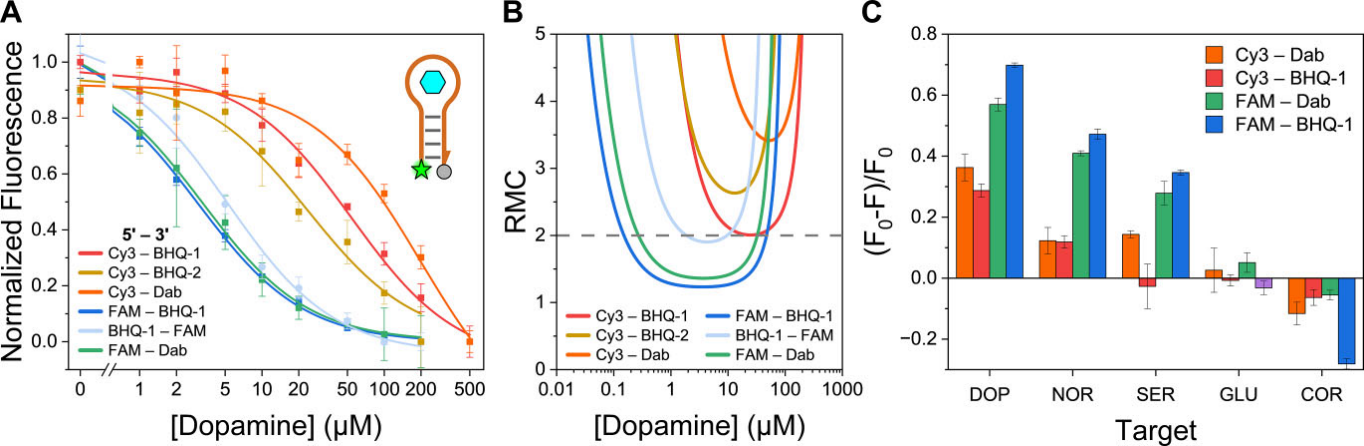
Impact of dye modification in a different aptamer switch architecture (MAB). (**A**) MAB aptamer-based switches with adjacent modification positions were evaluated by their affinities with varying pairs of fluorescent modifications. (**B**) RMC curves for each assay were calculated via equation (Eq. 3) from reference [39]. (**C**) MAB constructs’ specificity was assessed through subjection with 100 μM target: dopamine, norepinephrine, serotonin, glucose, or cortisol. Cy3 combinations possess markedly poorer affinity and resolution than FAM combinations, consistent with DBS trends, however, they exhibit superior specificity for dopamine. The relevant resolution parameters are listed in the Supplementary Note 3. The dashed line in panel (B) indicates the RMC cutoff at 2 (concentrations under the line are able to be statistically differentiated from a concentration twice as great). Data normalization and fits are explained in the “Materials and methods” section and in Supplementary Note 1. Plots were averaged over three replicates (*n* = 3) and error bars represent the standard deviation. Standard errors for equilibrium dissociation constants are listed in Supplementary Table S9.

To assess whether the observed trade-offs between affinity and kinetics due to the choice of the modification and its environment are unique to the DBS design or more broadly applicable, we extended our investigation to a different aptamer switch construct, the MAB. Using the same donor–acceptor combinations previously tested, we observed interesting results: Consistent with our earlier findings, combinations utilizing Cy3 again demonstrated weaker apparent binding affinity compared to the FAM-substituted ones (*K*_d_ = 53.8. ± 8.8, 12.4 ± 2.0, and 199.8 ± 88.5 μM for Cy3–BHQ-1, BHQ-2, and Dab, respectively; *K*_d_ = 3.0. ± 0.1 and 3.2 ± 0.2 for FAM–BHQ-1 and Dab, respectively) (Fig. 5A). While this generally mirrors the trends in the DBS, this information implies a decrease in affinity is due to either destabilizing interactions between donor and acceptor, and/or destabilizing effects of the modifications on stem formation (dye–DNA interactions) or the global aptamer structure (e.g. binding site). Furthermore, we tested Dab in this MAB system, and we found that the affinity diminished four-fold compared to the lowest affinity combination of Cy3–BHQ-1, suggesting even stronger destabilizing interactions. Lastly, we compared the impact of structural changes for BHQ by using BHQ-2 instead of BHQ-1, and the result showed that the affinity increased four-fold, demonstrating that changes in substituents on modifications with predominantly similar structure can have an impact on sensor affinity. We note that, unlike the DBS design, this system suffers from a more significant trade-off in resolution (Fig. 5B), potentially due to its “OFF” switching architecture, making the choice of fluorophore critical for any application requiring the ability to distinguish between small differences in concentration (Table 1, Supplementary Fig. S12, Supplementary Table S8, and Supplementary Note 3). Regarding kinetics, the change in fluorescence was faster than our instrument’s detection speed and thus could not be measured (Supplementary Fig. S13). Finally, we tested the specificity of all dopamine switches against biologically relevant molecules (i.e. norepinephrine and serotonin) and found it to be highly dependent on the identity of the fluorescent dye as well as its position (Fig. 5C and Supplementary Figs S14 and S15). Changing the fluorophore from Cy3 to FAM, or the position of Cy3–BHQ-1 from position 1 to 3, for instance, both significantly reduced specificity for dopamine. The Cy3-modified constructs were consistently more resilient to these specificity issues, underscoring the critical role of dye selection and positioning in sensor design.

**Table 1.** Performance summary of the impact of dye modification in the dopamine DBS and MAB

Architecture	Construct	*K*_d_ (μM)	*τ* (s)	T_m_ (°C)	Operating range (μM)*(fold change)
DBS(Apt–Dis)	Cy3–BHQ-1 (Position 1/Adjacent)	229.9 ± 22.0	995.0 ± 8.1	51.7 ± 0.1	9.2–620 *(68)
	FAM–BHQ-1 (Position 1)	6.1 ± 0.7	206.2 ± 0.8	49.1 ± 0.1	0.86–26 *(30)
	Cy3–BHQ-1 (Term.)	142.9 ± 11.7	565.7 ± 3.2	51.2 ± 0	5.6–590 *(110)
	Cy3–BHQ-1 (Int.)	34.0 ± 3.3	407.0 ± 1.1	50.9 ± 0.1	3.3–95 *(30)
	Cy3–BHQ-1 (Position 3)	2.3 ± 0.3	12.0 ± 0.4	50.3 ± 0.1	N/A
MAB(5′–3′)	Cy3–BHQ-1	53.8 ± 8.8	N/A	49.5 ± 0.3	N/A
	Cy3–Dab	199.8 ± 88.5	N/A	43.3 ± 1.0	N/A
	FAM–BHQ-1	3.0 ± 0.1	N/A	44.8 ± 0	0.15–47 *(310)
	FAM–Dab	3.2 ± 0.2	N/A	41.7 ± 0.2	0.27–32 *(119)

For a complete list of parameters, see Supplementary Tables S4, S5, S7–S9 in the Supplementary data. Data were averaged over three replicates (*n* = 3). Error for *K*_d_ and *τ* represents the standard error, and error for *T*_m_ represents the standard deviation.

To determine whether our findings were unique to the dopamine aptamer, we tested the impact of different fluorescent modifications on established sensor designs for other targets, such as ATP and serotonin (Supplementary Figs S16 and S17, and Supplementary Tables S10–S12). These experiments confirmed that the effects of such modifications are highly variable and specific to each aptamer system. For instance, contrary to our initial results, an ATP aptamer labeled with FAM showed a weaker affinity than one labeled with Cy3. Similarly, for a serotonin aptamer, changing the dye combination from FAM/TAM (*K*_d _≈ 120 nM) to Cy3/Cy5 effectively abolished binding.

### Discussion and future perspective

In this work, we show that when designing FRET-based aptamer switches, it is vital to consider the identity of fluorescent reporter pairs and their position within intramolecular-strand displacement-based systems. Our approach allows us to directly address a critical question in the design of FRET-based aptamer switches: Is it sufficient to develop fluorescent sensors based solely on photophysical principles, assuming that the specific fluorophore interactions and steric contributions have a negligible impact? Or, must we consider all these factors comprehensively to optimize these molecular tools for the application in question?

Importantly, we address a major misconception regarding the design of aptamer-based biosensors. To engineer aptamers into optical switches, structural alterations to the nucleic acid sequence (e.g. truncations, linker, or stem-loop additions) are often employed to facilitate reversible signaling [40, 41]. The resulting loss in apparent binding affinity is typically attributed solely to these sequence design changes, while the direct impact of the conjugated reporter molecules is often assumed to be minimal. In contrast, we demonstrate that these chemical modifications can be a primary source of affinity cost, diminishing binding properties by orders of magnitude relative to the parent aptamer. We showed experimentally, through using two different switch architectures and reporter pair combinations, that FAM exhibits significantly lower impact on sensor performance than Cy3 combinations, which in some cases can increase the effective *K*_d_ by >2 orders of magnitude. By measuring the thermal stability (i.e. apparent melting temperature, *T*_m_) of our different constructs, we found that higher stability generally correlated with weaker binding (Table 1 and Supplementary Table S13). However, the small differences in *T*_m_ are insufficient to explain the large affinity differences observed, which suggests that the underlying molecular interactions are more complex than simple stability effects [20–25]. This conclusion is consistent with previously published work on the impact of strand displacement length [26, 42]. This suggests the molecular interactions are more intricate than anticipated, possibly involving through-space effects (e.g. electrostatics), structural propagation, or direct interference with hairpin binding dynamics.

To mitigate the affinity cost associated with reporter modifications on aptamer switches, we have explored several strategies. We first demonstrated that introducing a spatial offset—initially by one nucleotide—between the fluorophore and quencher can improve apparent binding affinity, likely by eliminating dye–DNA or direct interactions between the pair. While this approach can increase affinity, it must be balanced against potential losses in quenching efficiency and specificity. Second, we showed that evaluating sensor performance beyond theoretically optimal FRET positions is critical to mitigate cost on affinity. We demonstrated that a less favorable FRET distance change can be compensated for by a more optimal local reporter environment and spacer flexibility, which enhanced the apparent binding affinity by a factor of 100. This gain in affinity, however, was achieved with a concomitant reduction in the sensor’s operating range and reduction in specificity, revealing a key performance trade-off. This demonstrates the critical importance of selecting a reporter pair by prioritizing the specific parameters and achieving the optimal balance of affinity, specificity, and dynamic range that matter most for the particular assay in question.

For this study, we selected the most ubiquitous reporters to examine; however, the selections are by no means exhaustive. Future research can more comprehensively investigate different dye classes to correlate structural properties and DNA-binding type (i.e. intercalating or groove binding) to sensor performance. In particular, there is interest in evaluating the performance of anionic sulfo-Cy3 in relation to cationic Cy3 to determine whether the change in charge will mitigate Cy3’s impact on sensor performance while retaining its spectral properties, as our research and literature suggest [17]. One can also explore alternative strategies to regulate reporter pair interactions, such as investigating the effects of chemical spacer length, rigidity, and dye solubility [43–46]. This builds upon existing literature that has demonstrated the influence of dye hydrophilicity and the destabilizing effect of certain reporters on DNA duplex stability [47–50]. A primary goal is to enable the placement of reporters to maximize signal while minimizing structural perturbations. Future studies must elucidate the precise mechanisms by which reporter modifications alter aptamer sensor performance. Distinguishing whether these effects stem from dye–DNA, dye–dye, or dye–target interactions is challenging and requires advanced characterization techniques such as isothermal titration calorimetry [27], nuclear magnetic resonance spectroscopy [51], and computational modeling [52, 53].

The main challenge is the lack of generalizability, as a comprehensive model for one aptamer cannot be adopted for a different one. In fact, we showed in this work that key performance metrics—such as apparent equilibrium affinity (*K*_d_), kinetics, specificity and operating range—can be decoupled, not generalizable and highly design- and target-dependent. Hence, the influence of these modifications on sensor performance, is largely unpredictable based on current models. To this end, established high-throughput screening approaches could be adapted not only to select the best binder in terms of sequence and library design, but also to co-optimize the best-performing fluorophore pair for that specific design [54, 55]. A rational design in this context would not be about predicting the best dye and position from first principles. Instead, it involves implementing a screening workflow early in the process to systematically evaluate key variables, thereby reducing the intensive trial-and-error workload later. This rational screening should account for the modification’s position, the identity of the fluorophore–quencher pair, their proximity to each other, the overall sensor architecture, and the aptamer’s selection history and binding mechanism.

Ultimately, given the significant cost and complexity of optimizing labeled systems, label-free technologies represent a compelling long-term alternative. Future work can also focus on developing aptasensor platforms based on advanced platforms like optical microring resonators [56], silicon nanowire field-effect transistors [19], and sophisticated plasmonic sensors [57]. However, the success of these approaches is fundamentally contingent on advancements in materials science (for anti-fouling surfaces) [58], molecular engineering (for better aptamers) [26, 29], and system design (for noise cancellation) [59]. Success in these areas can enable the next generation of label-free diagnostics.

## Supplementary Material

gkaf1346_Supplemental_Files

## Data Availability

All data underlying the findings of this study are available from the authors upon request. The source data underlying figures and, Supplementary figures and tables are provided as a “Source data” file.
