# Supplementary material for: Fluorescent modifications in aptamer switches—positional, structural, and neighboring pair effects on sensor performance
Source: Nucleic Acids Res. 2025 Dec 12;53(22):gkaf1346. doi: 10.1093/nar/gkaf1346 (PMC12700101; doi:10.1093/nar/gkaf1346)
Supplement: gkaf1346_Supplemental_Files [file gkaf1346_supplemental_files.zip › Supplementary_Data_file_UpdatedSXcallouts.docx]

**Supplementary Information**

Fluorescent Modifications in Aptamer Switches – Positional, Structural, and Neighboring Pair Effects on Sensor Performance.

Albert Zehan Li^1^, Conrad H. T. Belzberg^1^, Mimansa Sidhu, Amani A. Hariri^*^

^1^equal contribution, *Corresponding author

**Outline**

**Figures**

**Supplementary Figure S1:** Structures of fluorescent modifications used and their chemical linkage to DNA

**Supplementary Figure S2:** Sample Raw concentration-dependent fluorescence emission spectra of DBS and MAB binding curves

**Supplementary Figure S3:** Dopamine-dependent fluorescence signal change of fluorophore-labeled aptamer strands.

**Supplementary Figure S4:** Time-dependent fluorescence of dopamine-construct binding curves

**Supplementary Figure S5:** Sample Raw kinetic traces of DBS.

**Supplementary Figure S6:** Sample melting curves and data processing.

**Supplementary Figure S7:** Performance heatmaps of dopamine DBS and MAB of varying modification pairs

**Supplementary Figure S8:** Minimum free energy structure of DBS with and without thymidine insertion (NUPack)

**Supplementary Figure S9:** Impact of fluorophore-quencher strand ratio on the sensor performance of the Cy3–BHQ-1 DBS

**Supplementary Figure S10:** Comparison between apparent affinity and temporal response for DBS constructs with varying fluorophore–quencher

**Supplementary Figure S11:** Binding curves for Cy3/Cy5 FRET design at different excitation and emission wavelengths.

**Supplementary Figure S12:** Resolution of molecular concentration of DBS with different modification combinations, strand ratios, and offsets

**Supplementary Figure S13:** Kinetic traces of MAB with varying reporter pair

**Supplementary Figure S14:** Impact of modification identity and position on DBS target specificity.

**Supplementary Figure S15:** Target-dependent signal increase of fluorophore-labeled dopamine aptamer and DBS (controls).

**Supplementary Figure S16:** Impact of modification identity on sensor affinity for the ATP intramolecular strand displacement (ISD) sensor.

**Supplementary Figure S17:** Impact of modification identity and position on sensor affinity for the dual-labeled Serotonin aptamer sensor and Serotonin DBS.

**Tables**

**Supplementary Table S1:** DNA sequences used for the DBS and MAB systems

**Supplementary Table S2:** Legend of modifications for sequences used in this work

**Supplementary Table S3:** Apparent equilibrium dissociation constants for Cy3 – BHQ-1 MAB and FAM – BHQ-1 DBS over time

**Supplementary Table S4:** Apparent equilibrium dissociation and time constants for DBS with varying fluorescent reporter pair combinations

**Supplementary Table S5:** Apparent equilibrium dissociation and time constants for DBS using Cy3 and BHQ-1 with varying offsets

**Supplementary Table S6:** Apparent equilibrium dissociation and time constants for DBS using Cy3 and BHQ-1 varying aptamer:displacement ratios

**Supplementary Table S7:** Apparent equilibrium dissociation and time constants using Cy3 – BHQ-1 with varying positions

**Supplementary Table S8:** Sensor metrics for modification combinations and different sensor architectures

**Supplementary Table S9:** Apparent equilibrium dissociation constants for MAB with varying fluorescent reporter pair combinations

**Supplementary Table S10:** Apparent equilibrium dissociation constants for ATP-ISD with varying fluorescent reporter pair combinations

**Supplementary Table S11:** Apparent equilibrium dissociation constants for dual-labeled serotonin sensor with varying fluorescent reporter pair combinations

**Supplementary Table S12:** Apparent equilibrium dissociation constants for serotonin DBS with varying fluorescent reporter pair positions

**Supplementary Table S13:** Apparent melting temperatures for constants DBS and MAB with varying fluorescent reporter pair combinations and positions

**Notes**

**Supplementary Note 1:** Method of analysis for apparent binding affinity and observed kinetics of aptamer-switch sensors

**Supplementary Note 2:** Estimating degrees of freedom/motion: Angle range at which FRET is prevalent in DBS system at both terminal and internal positions (positions 1, 2 and 3)

**Supplementary Note 3:** Comparison of sensor metrics – Resolution of molecular concentration, limit of detection, operating range, and sensitivity.

**References**

**Supplementary Figures**

**
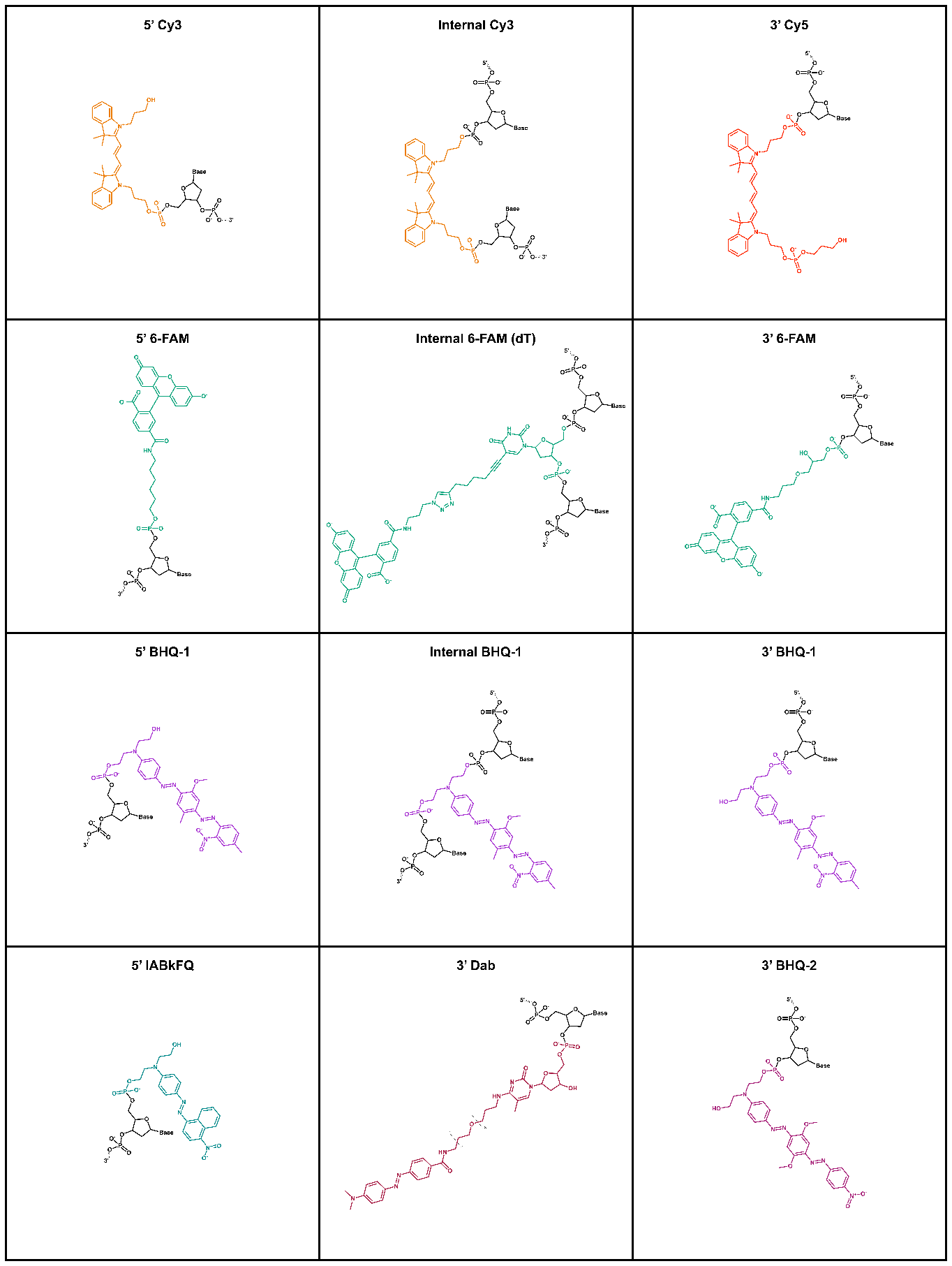
**

**Supplementary Figure S1.** **Structures of fluorescent modifications used and their chemical linkage to DNA.** Note: IDT incorporates internal 6-FAM on a thymidine, internal Cy3 in the phosphate backbone, and 3’ Dab on a 5-methyldeoxycytidine. Structures of Iowa Black FQ are based on published structures^1^, and proposed bioconjugation is informed using publicly available molecular weights.


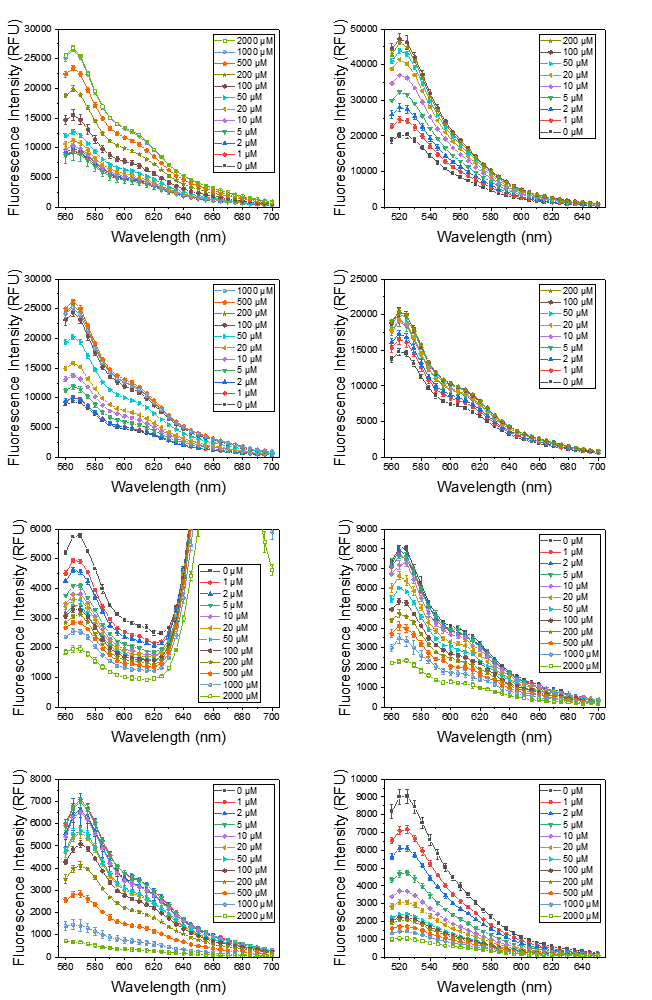


**A**

**B**

**C**

**D**

**E**

**F**

**G**

**H**

**Supplementary Figure S2.** **Sample Raw concentration-dependent fluorescence emission spectra of DBS and MAB binding curves.** DBS: (A) Cy3– BHQ-1 (Position 1). (B) FAM – BHQ-1. (C) Cy3 – BHQ-1 (Internal Offset). (D) Cy3 – BHQ-1 (Position 3). (E) Cy3/Cy5 – No. 1:4 aptamer:displacement strand ratio. MAB: (F) Cy3 – BHQ-1. (G) Cy3 – Dab. (H) FAM – BHQ-1 The fluorescence at peak emission was used as raw signal for thermodynamic and kinetic studies (See **Materials and Methods Section**). All plots show averages over three replicates (n= 3).


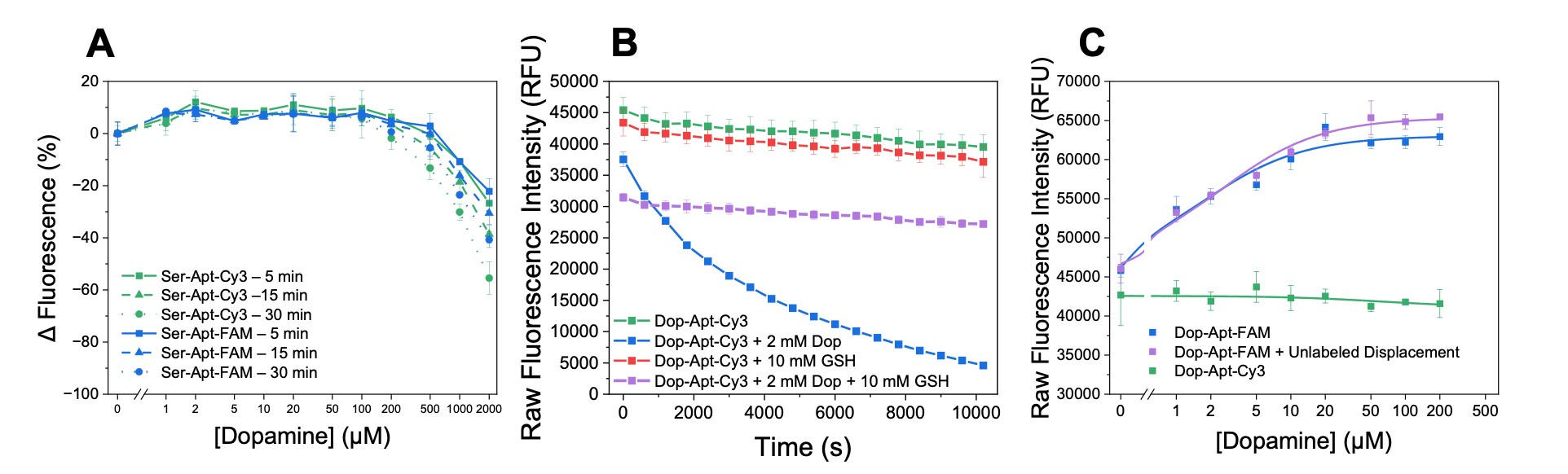


**Supplementary Figure S3. Dopamine-dependent fluorescence signal change of fluorophore-labeled aptamer strands.** (A) Dopamine-mediated fluorescence decreases of Cy3 and FAM-labeled serotonin aptamer strands at 5-,15-, and 30-min target incubation at 25 °C. (B) Time-dependent dopamine-mediated fluorescence decrease of Cy3-labeled dopamine aptamer strand. L-glutathione (GSH) is a known antioxidant and reduces dopamine oxidation/polydopamine formation, as demonstrated by the significant reduction of fluorescence decrease (purple line), even after 2.5 hours.^2^ (C) Collective target and sequence-dependent fluorescence increase of FAM-labeled dopamine aptamer strand. FAM-labeled dopamine aptamer strand in isolation or with a quencher absent displacement strand, exhibits considerable fluorescence increase (*K*_d_ = 1.7 ± 0.5 and 2.4 ± 0.4 μM, respectively, presumably due to a FAM-sequence specific environmental effect. Error bars are standard deviations of triplicate measurements. Control sequences used in (A) are derived from Nakatsuka *et al*., 2018 *as follows: Cy3-labeled serotonin aptamer sequence with anchor (Ser-Apt-Cy3): AAA TAC AAC AAG AAA AAA /iCy3/CT CTC GGG ACG ACT GGT AGG CAG ATA GGG GAA GCT GAT TCG ATG CGT GGG TCG TCC C. FAM-labeled serotonin aptamer sequence (Ser-Apt-FAM): /56-FAM/CT CTC GGG ACG ACT GGT AGG CAG ATA GGG GAA GCT GAT TCG ATG CGT GGG TCG TCC C.*^3^ Sequences used in (B) and (C) were from Hariri *et al.*, 2024 *as follows: Cy3 or FAM-labeled dopamine aptamer sequence with anchor and linker (Dop-Apt-Cy3/Dop-Apt-FAM): AAA TAC AAC AAG AAA AAA CTC TCG GGA CGA CGXCCA GTT TGA AGG TTC GTT CGC AGG TGT GGA GTG ACG TCG TCC C, where X is iCy3 or i6-FAMK. Dopamine DBS displacement sequence (Unlabeled Displacement): CGT CGT CCC CTC TTC ACA TAT CTA TTT TTT CTT GTT GTA TTT/3Bio/.*^4^


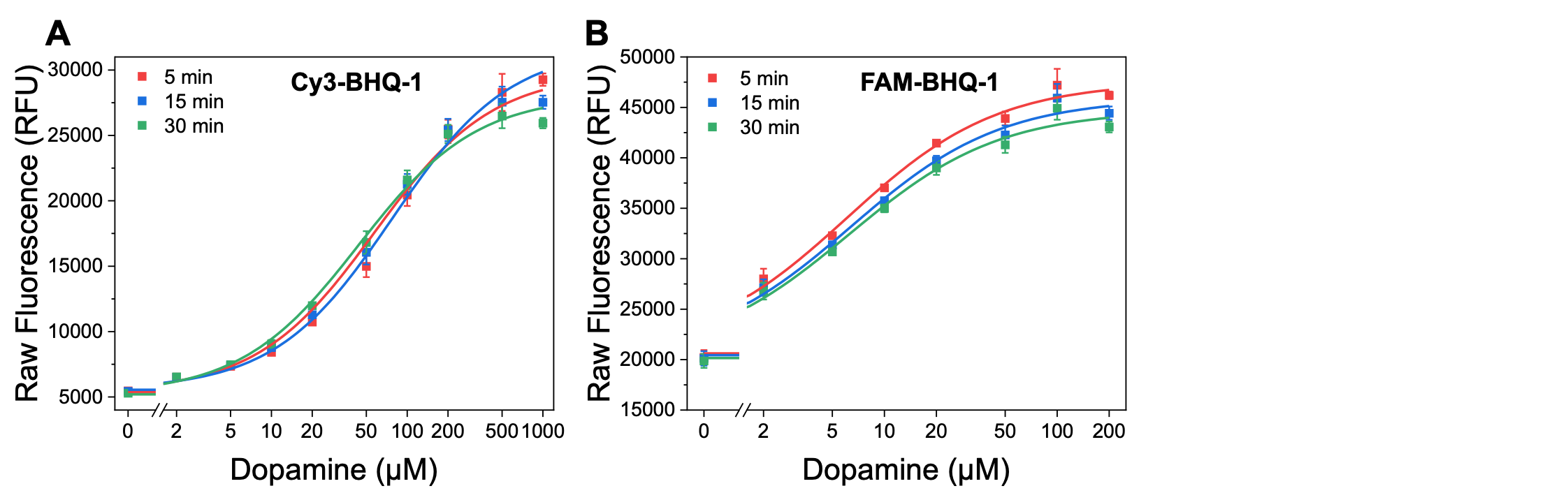


**Supplementary Figure S4. Time-dependent fluorescence of dopamine-construct binding curves.** Binding curves of (A) Cy3 – BHQ-1 (MAB) and (B) FAM – BHQ-1 (DBS) at position 1 resulting from a 5-, 15-, or 30-min incubation at 25 °C. All plots are averaged over three replicates (n = 3). Error bars represent the standard error of the average. Data fitting is explained in the **Materials and Methods Section** and in **Supplementary Note 1**. Plots were averaged over three replicates (n = 3) and error bars represent the standard deviation. Standard errors for equilibrium dissociation constants are listed in **Supplementary Table S3**.


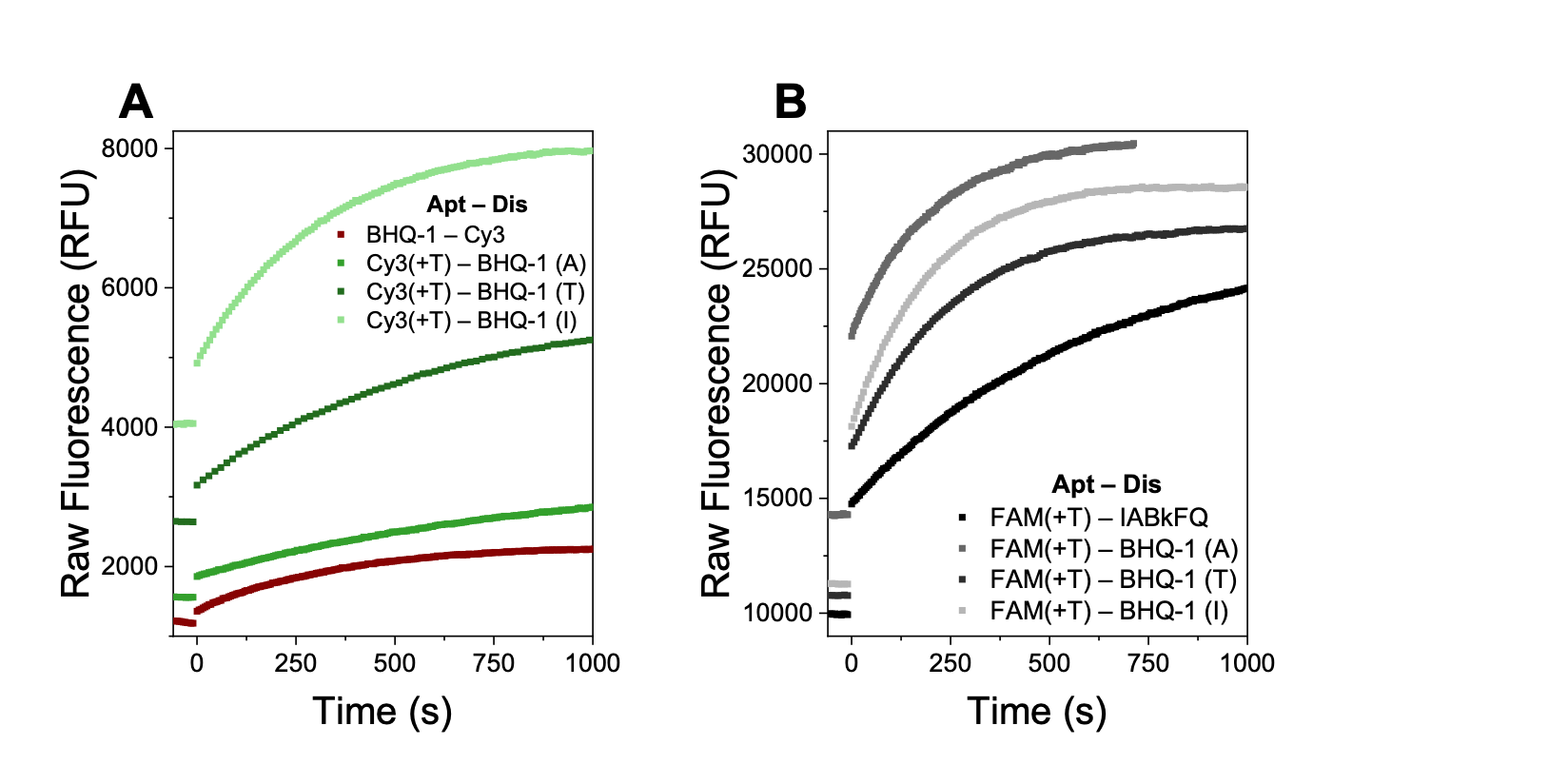


**Supplementary Figure S5.** **Sample Raw kinetic traces of DBS.** Constructs were pre-incubated for at least 1 min, followed by the addition of 25 μM dopamine. (A) Raw kinetic traces of Cy3-labelled constructs. (B) Raw kinetic traces of FAM-labelled constructs. Data fitting is explained in the **Materials and Methods Section** and in **Supplementary Note 1**. Plots were averaged over three replicates (n = 3) and error bars were omitted for clarity. Standard errors for time constants are listed in the relevant **Supplementary Tables S4 and S5**.

**
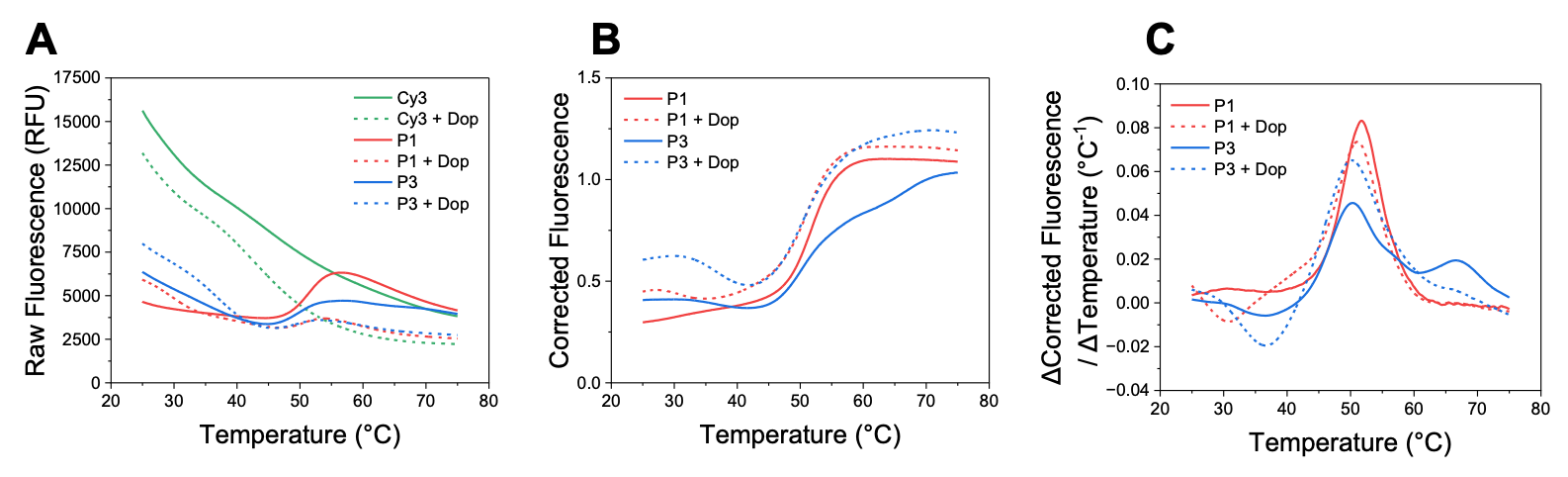
**

**Supplementary Figure S6.** **Sample melting curves and data processing.** (A) Raw melting curve data of the Cy3-labeled dopamine DBS aptamer strand and the Cy3 – BHQ-1 construct at positions 1 (P1) and 1 (P2), in absence and presence of 50 µM dopamine. Cy3 exhibits a temperature dependent fluorescence intensity, deflating the fluorescence increase of the DBS constructs. (B) Melting traces were normalized (corrected) by the corresponding fluorophore or fluorophore and dopamine control traces. (C) First derivative plot of corrected melting curve traces. Data correction and normalization is explained in the **Materials and Methods Section**. Plots were averaged over three replicates (n = 3) and error bars were omitted for clarity. Standard deviations for melting temperatures are listed in the **Supplementary Table S13**.

**A**

**B**

**C**

**Supplementary Figure S7.** **Performance heatmaps of dopamine DBS and MAB of varying modification pairs**. (A) DBS affinity and (B) kinetics of varying modification pairs, and (C) MAB affinity of varying modification pairs. All plots are averaged over three replicates (n = 3). Data normalization and fits are detailed in the **Materials and Methods Section** and **Supplementary Note 1**. Sample raw thermodynamic plots and relevant parameters for the constructs are provided in **Supplementary Figure S2** and **Supplementary Tables S4 and S9**.

**
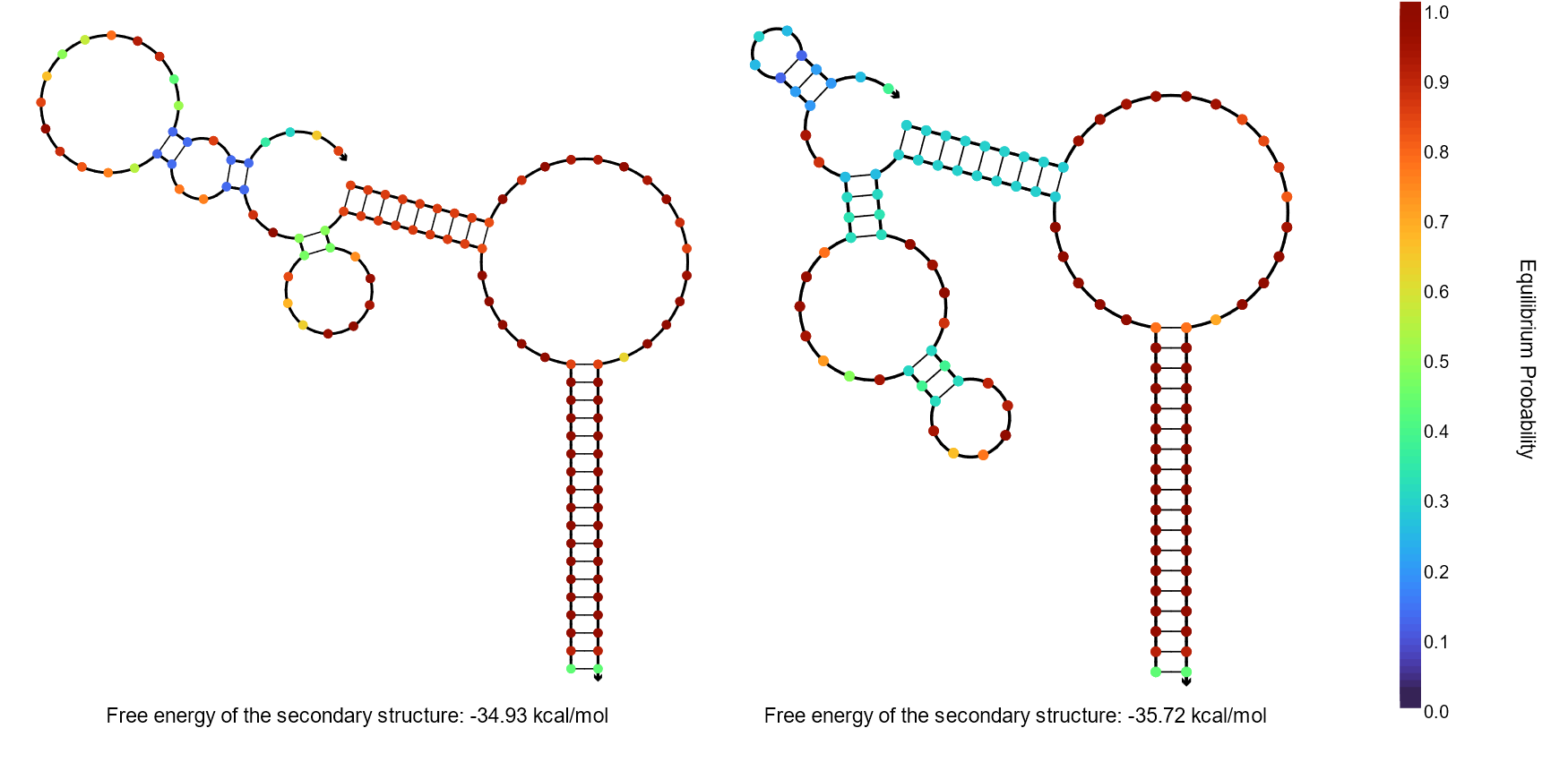
**

**B**

**A**

**Supplementary Figure S8. Minimum free energy structure of DBS with and without thymidine insertion (NUPack)**. (A) No thymidine insertion. (B) with thymidine insertion. NUPack parameters: DNA (Material), 25 °C (Temperature), DNA – dna04.1 (Parameters), All stacking (Ensemble), Na^+^ – 0.15 M, Mg^++^ – 0.002 M (Salts), 1 μM: 4 μM (aptamer:displacement strand ratio), 4 strands (Max complex size).

**
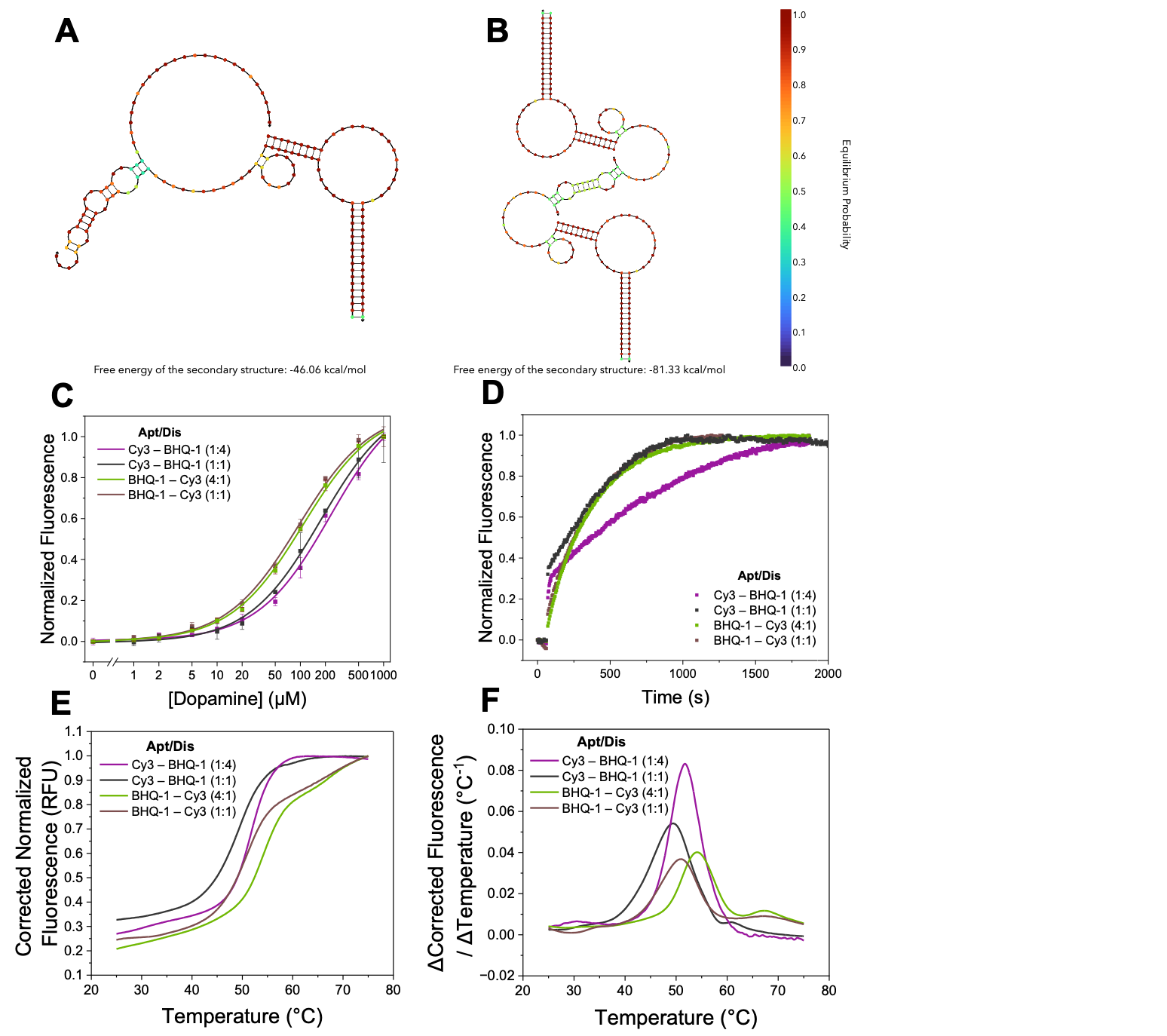
**

**Supplementary Figure S9. Impact of fluorophore–quencher strand ratio on the Cy3–BHQ-1 DBS sensor performance.** Potential minimum free energy structure of DBS at different ratios 1:1 and 1:4 (NUPack) (A) aptamer-displacement-displacement or (B) aptamer-displacement-aptamer-displacement species. (C) Binding curves and (D) kinetics traces for varying Cy3 – BHQ-1 strand ratios. (E) Normalized corrected melting curve traces and (F) first derivative plots for varying Cy3 – BHQ-1 strand ratios. All plots are averaged over three replicates (n = 3). Error bars in (C) represent the standard deviation of the average. Error bars were removed in (D) for clarity. Data normalization and fits are detailed in the **Materials and Methods Section** and in **Supplementary Note 1**. Sample raw thermodynamic plots and relevant parameters for the constructs are provided in **Supplementary Figure S2** and **Supplementary Tables S6 and S13**. NUPack parameters: DNA (Material), 25 °C (Temperature), DNA – dna04.1 (Parameters), All stacking (Ensemble), Na+ – 0.15 M, Mg++ – 0.002 M (Salts), 1 μM: 4 μM (aptamer:displacement strand ratio), 4 strands (Max complex size).


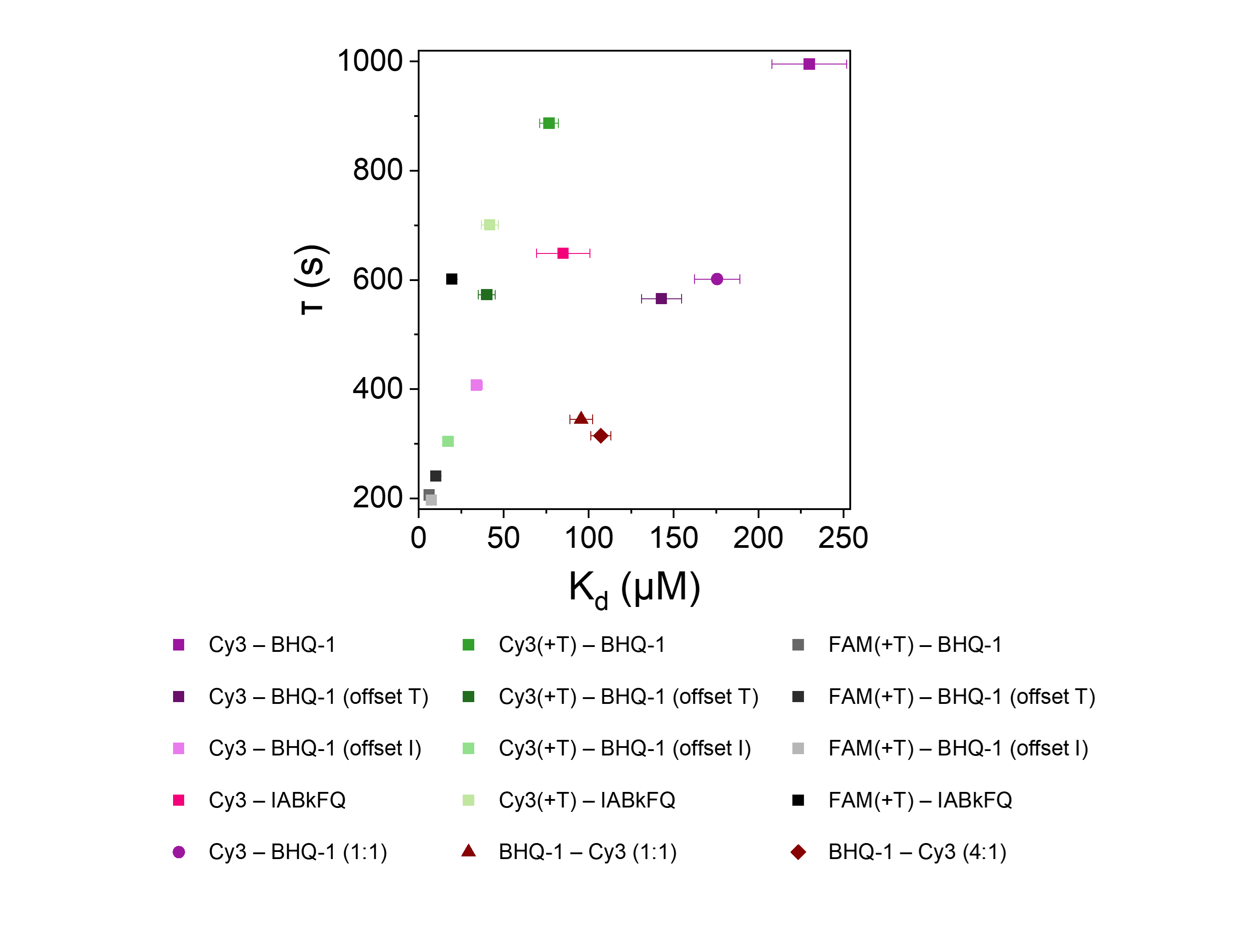

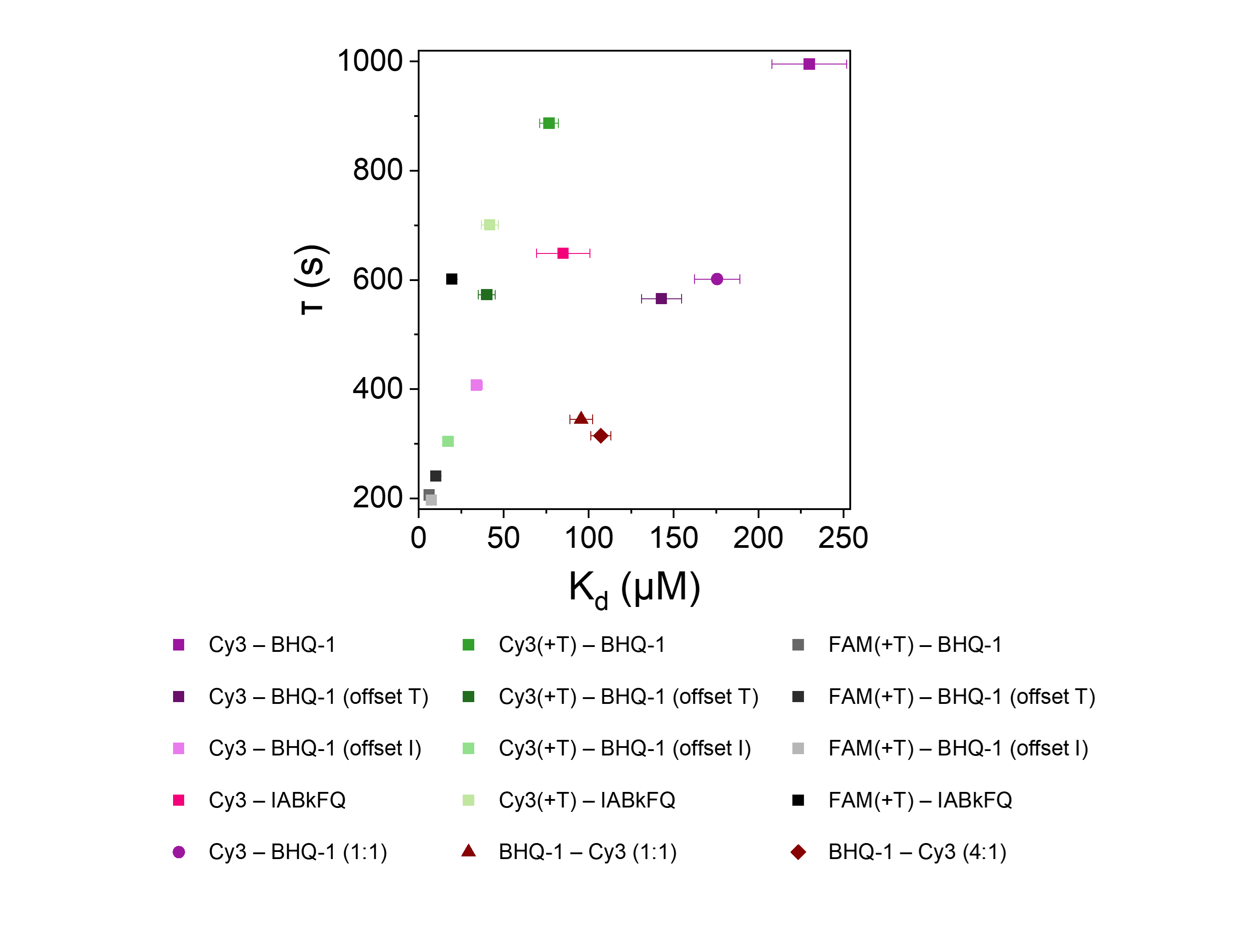

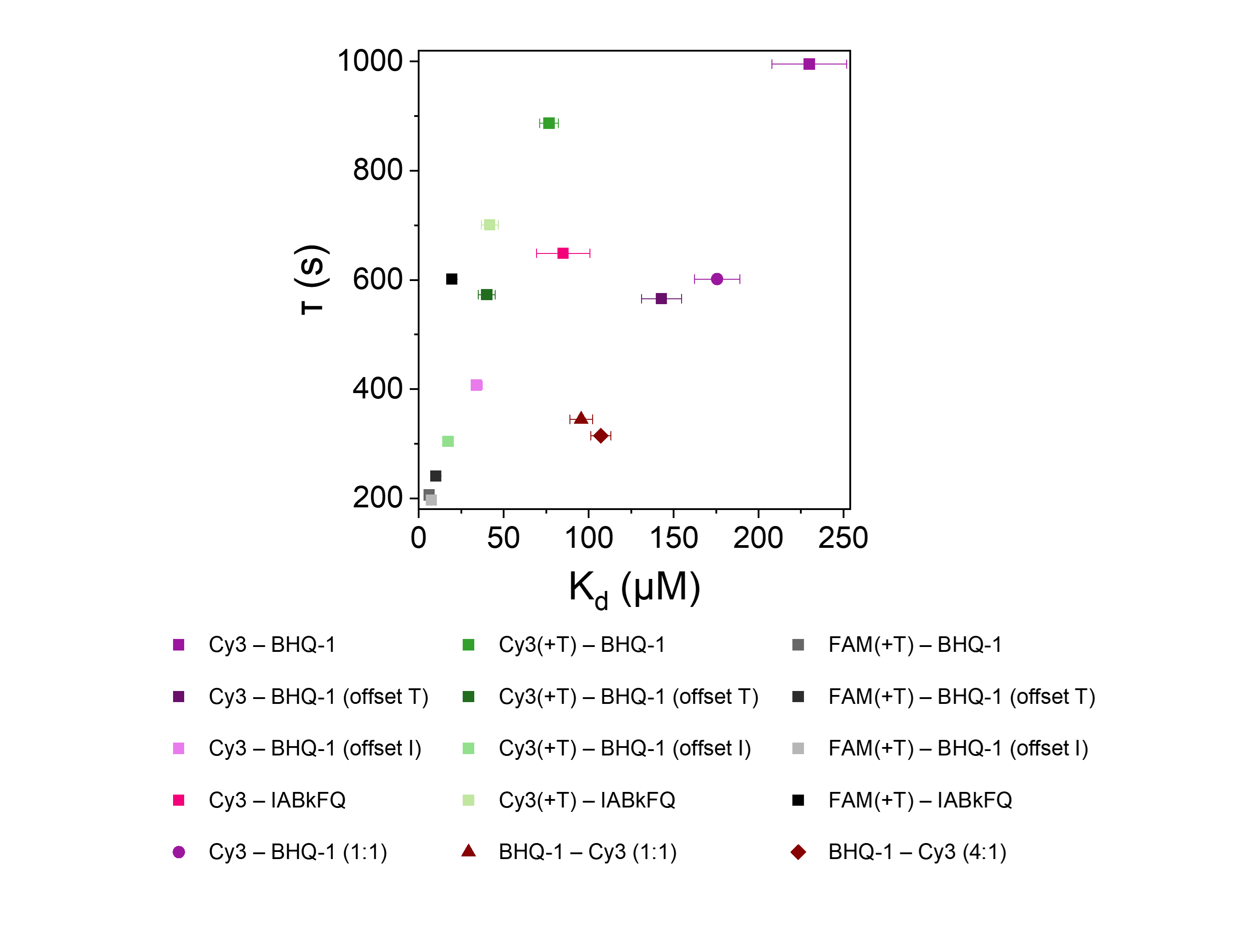


**A**

**B**

**Supplementary Figure S10. Comparison between apparent affinity and temporal response for DBS constructs with varying fluorophore** – **quencher pairs**. (A) Modification pairs with similar affinities can have vastly different kinetics, and conversely, (B) pairs with similar kinetics can exhibit markedly different affinities. All plots are averaged over three replicates (n = 3). Error bars in represent the standard error. Data normalization and fits are detailed in the **Materials and Methods Section** and in **Supplementary Note 1**. Sample raw thermodynamic plots and relevant parameters for the constructs are provided in **Supplementary Figure S2** and **Supplementary Tables S4, S5, and S6**.


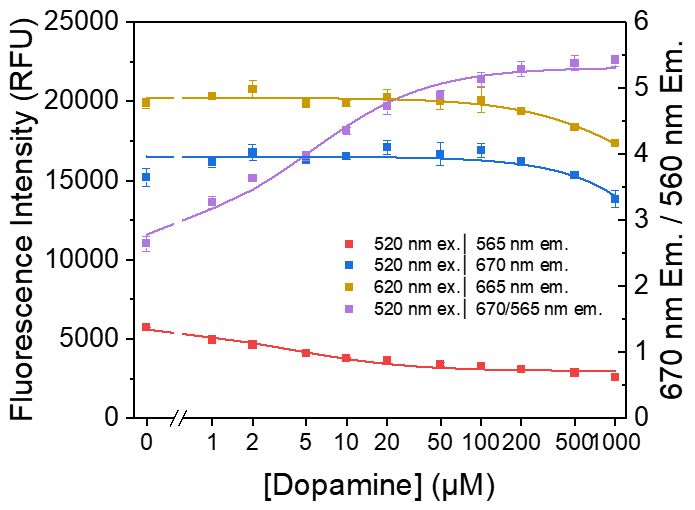


**Supplementary Figure S11.** **Binding curves for Cy3/Cy5 FRET design at different excitation and emission wavelengths.** Left axis: Excitation at 520 and 620 nm and plotting the raw fluorescence emission at 670 and 665 nm, respectively, does not produce any signal change, presumably due to increases in Cy5 emission being balanced by quenching with the new local environment (after target binding). Right axis: Excitation at 520 nm and plotting the raw fluorescence emission at 565 nm or the ratio of 670/565 nm produces a signal change. Data normalization and fits are explained in the **Materials and Methods Section** and in **Supplementary Note 1**. Plots were averaged over three replicates (n = 3). Error bars represent the standard deviation.


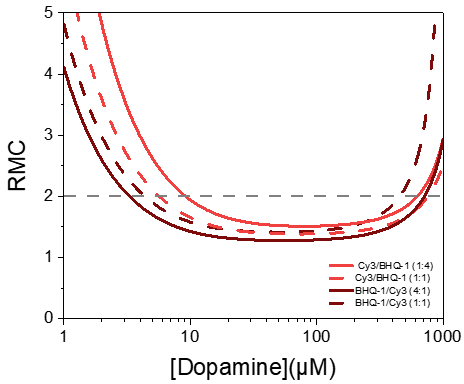

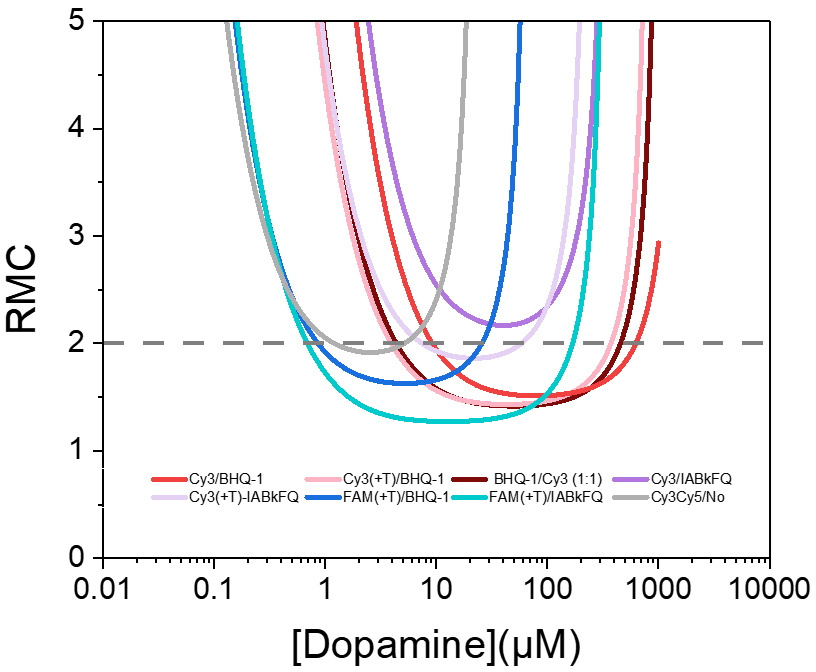


**B**

**A**


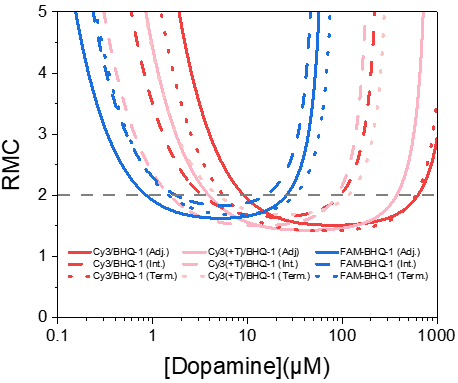


**C**

**Supplementary Figure S12. Resolution of molecular concentration of DBS with different modification combinations, strand ratios, and offsets.** For the DBS, there are no significant differences in the minimum RMC and the breadth of operating range for different (A) DBS modification combinations, (B) DBS Cy3 **–** BHQ-1 strand ratios, and (C) DBS offsets. Dashed line in grey represents the operating range cutoff: RMC < 2. Resolution of molecular concentration is calculated as described in **Supplementary Note 3**.


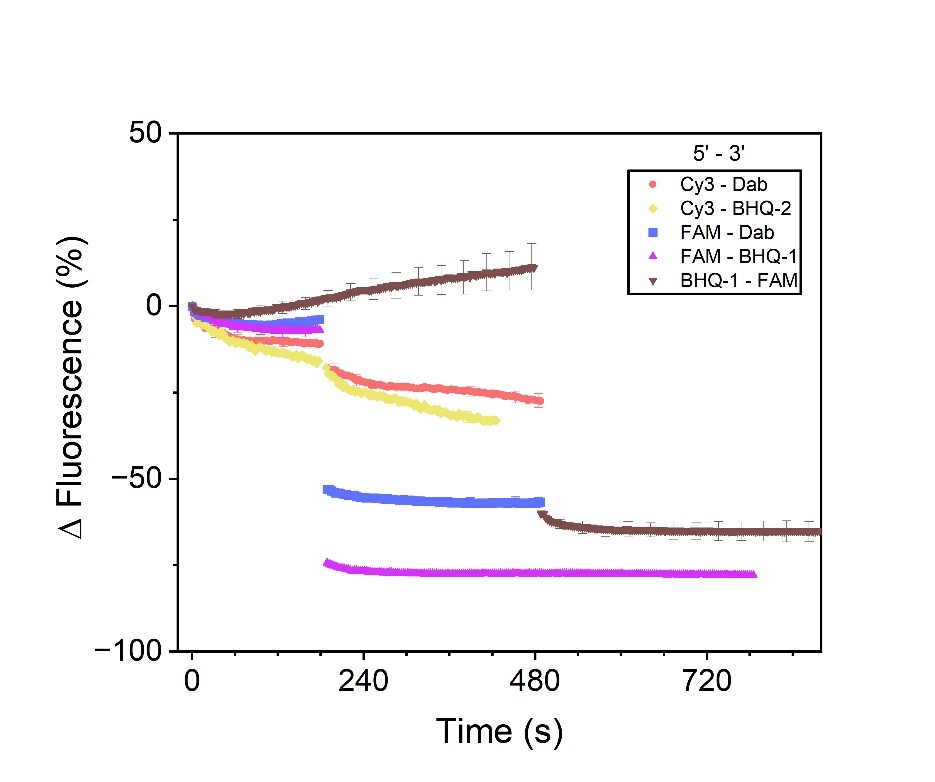


**Supplementary Figure S13.** **Kinetic traces of MAB with varying reporter pair**. Constructs were pre-incubated for 180 s, followed by the addition of 25 µM dopamine (red arrow) for all combinations (except BHQ-1 – FAM at 480 s). Error bars are standard deviations of triplicate measurements (every 10 data points for clarity). Data normalization and fits are detailed in the **Materials and Methods Section** and in **Supplementary Note 1.**


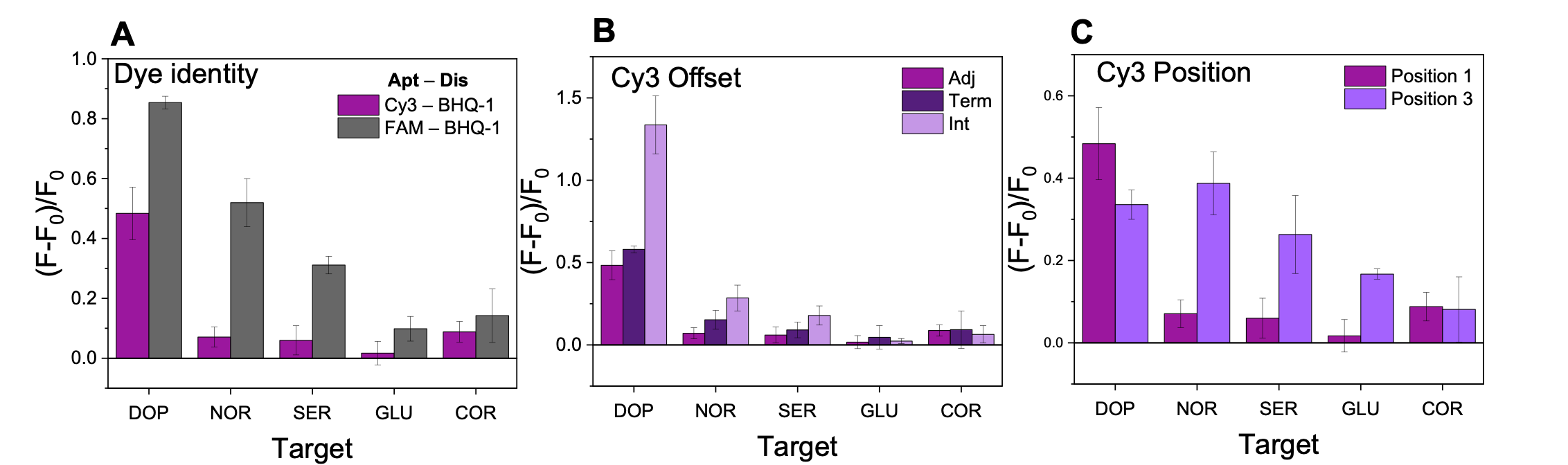


**Supplementary Figure S14.** **Impact of modification identity and position on DBS target specificity.** Constructs probing the influence of (A) identity (Cy3 – BHQ-1 and FAM – BHQ-1 at position 1), (B) offset position (Cy3 – BHQ-1 at the adjacent, terminal and internal offset positions), and (C) local DNA environment (Cy3 – BHQ-1 at positions 1 and 3) were subjected to 100 μM target; dopamine, structurally related targets norepinephrine (NOR) and serotonin (SER), or environmentally relevant targets glucose (GLU) and cortisol (COR). FAM-labeled DBS and Cy3 – BHQ-1 at position 3 exhibited significantly worse specificity compared to Cy3 – BHQ-1 at position 1. There was no significant difference in specificity within the different Cy3 – BHQ-1 offset positions. All plots are averaged over three replicates (n = 3). Error bars represent the standard deviation of the average. Data normalization is detailed in the **Materials and Methods Section.**


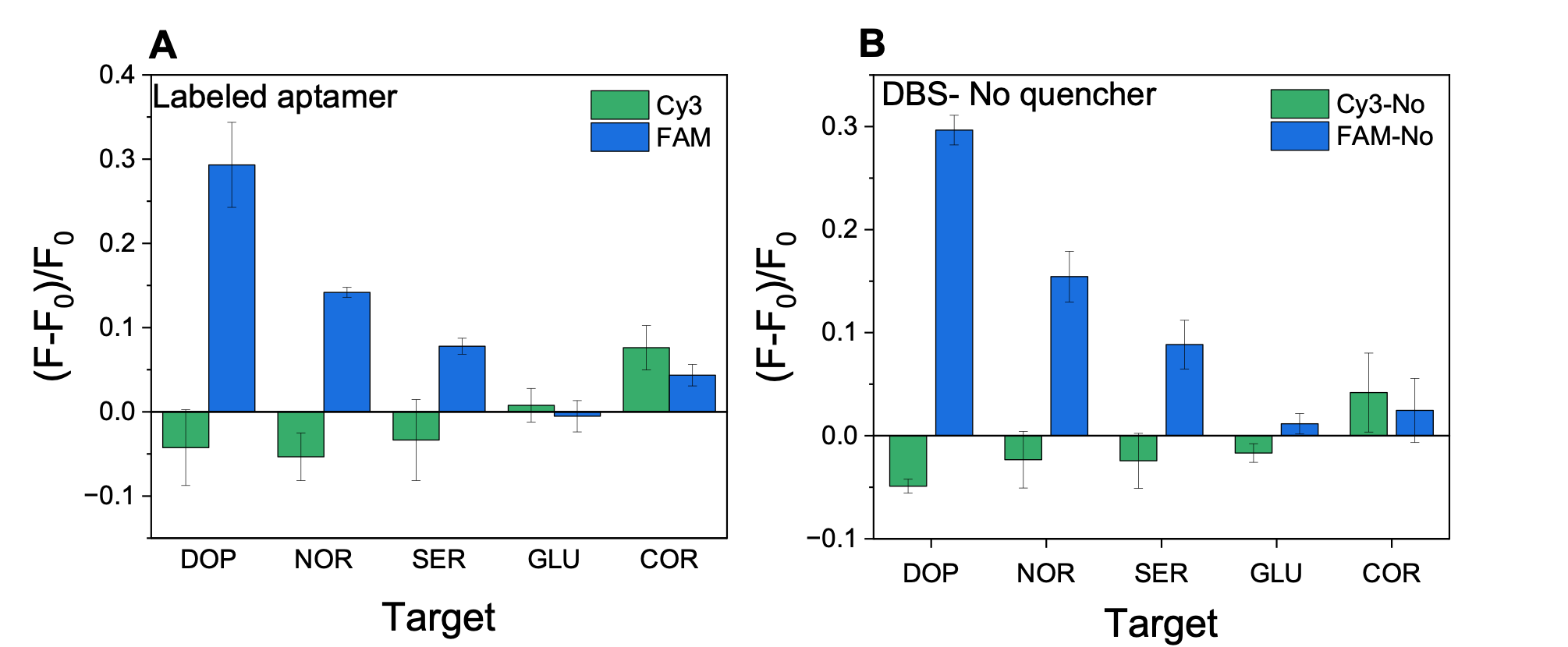


**Supplementary Figure S15.** **Target-dependent signal increase of fluorophore-labeled dopamine aptamer and DBS (controls).** Cy3 or FAM-labeled (A) dopamine aptamer strand or (B) DBS without quencher on the displacement strand were subjected to 100 μM target: dopamine (DOP), norepinephrine (NOR), serotonin (SER), glucose (GLU), or cortisol (COR). Cy3-labeled aptamer strand or DBS without quencher does not exhibit any target-dependent fluorescence increase, however the same FAM-labeled constructs produce a significant signal increase in the presence of dopamine and structurally similar targets norepinephrine and serotonin. All plots are averaged over three replicates (n = 3). Error bars represent the standard deviation of the average. Data normalization is detailed in the **Materials and Methods Section.**


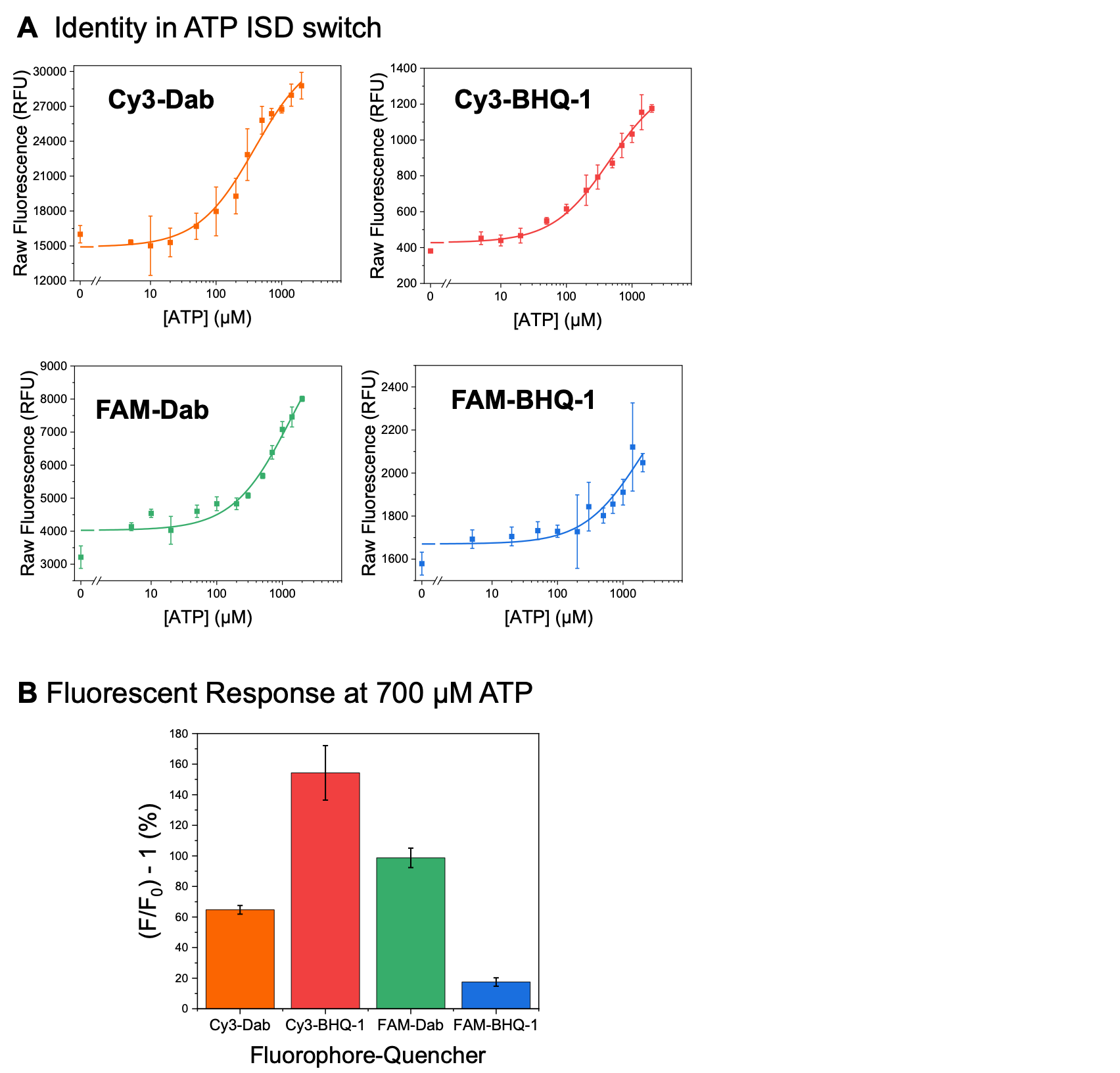


**Supplementary Figure S16. Impact of modification identity on sensor affinity for the ATP intramolecular strand displacement (ISD) sensor.** (A) Binding curves for the ATP ISD with varying fluorophore-quencher pairs (5’ – 3’): Cy3 – Dab, Cy3 – BHQ-1, FAM – Dab, and FAM – BHQ-1. (B) Plot of the fluorescence response of each construct at 700 µM ATP. Data fitting is explained in the **Materials and Methods Section** and in **Supplementary Note 1**. Plots were averaged over three replicates (n = 3) and error bars represent the standard deviation. Standard errors for equilibrium dissociation constants are listed in **Supplementary Table S10**. Sequence used was the 25-7 ATP ISD, *as follows: XCA CCT GGG GGA GTA TTG CGG AGG AAG GTT TTT CCA GGT GY, where X is 5Cy3 or 56-FAM and Y is 3DAb or 3BHQ_1.*^5^


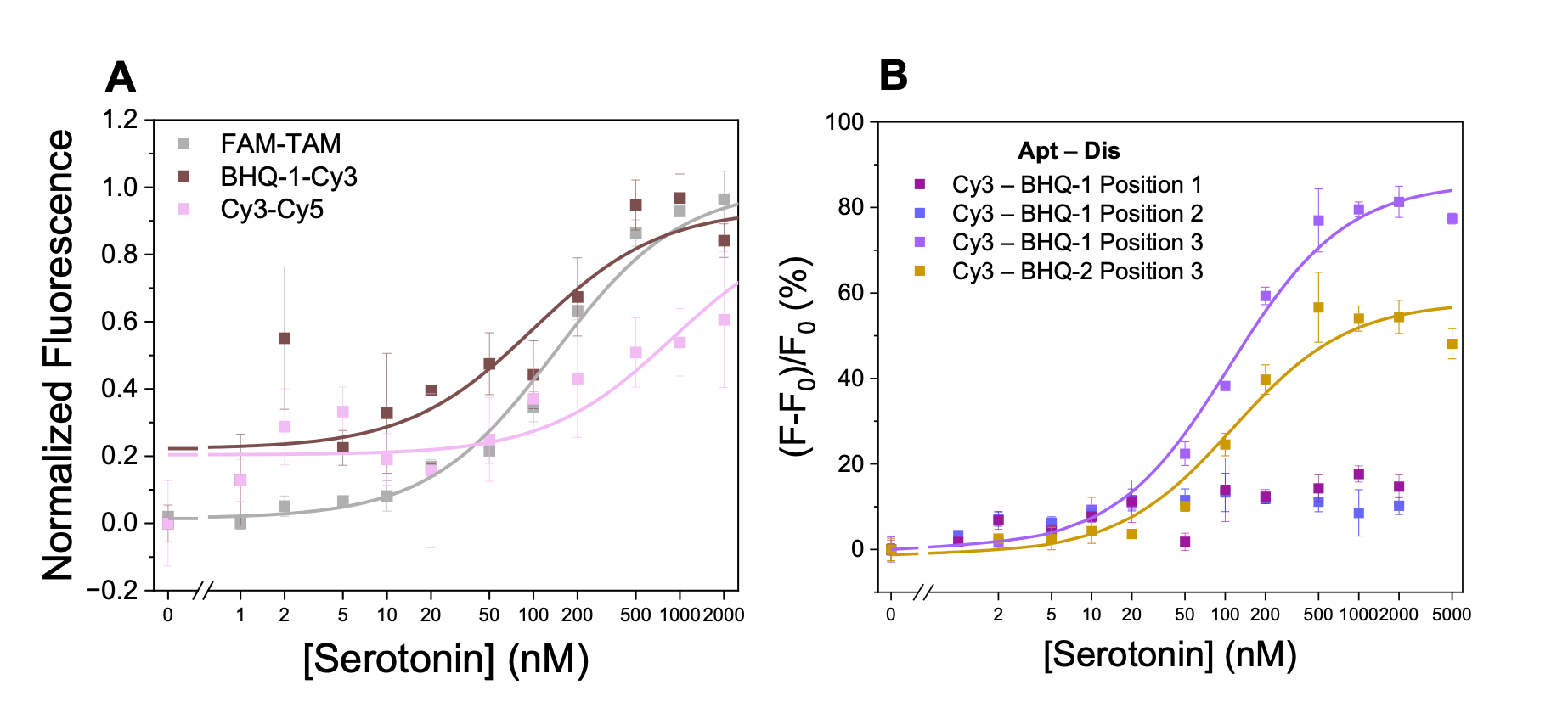


**Supplementary Figure S17. Impact of modification identity and position on sensor affinity for the dual-labeled Serotonin aptamer sensor and Serotonin DBS.** Binding curves for (A) the dual-labeled serotonin aptamer sensor with varying fluorescent modification pairs and (B) the serotonin DBS with Cy3 – BHQ-1 (or BHQ-2) at positions 1, 2, or 3. For the serotonin sensor, there was an insignificant difference in apparent affinity) for the FAM-TAM (Kd = 120 nM) and BHQ-1-Cy3 (Kd = 100 nM), however both were two orders of magnitude better than the Cy3-Cy5 labeled sensor (Kd = 8900 nM). For the serotonin DBS, only the modification pair at position 3 resulted in a functional switch, while binding was effectively abolished at positions 1 and 2. All plots are averaged over three replicates (n = 3). Error bars represent the standard error of the average. Data fitting is explained in the **Materials and Methods Section** and in **Supplementary Note 1**. Plots were averaged over three replicates (n = 3) and error bars represent the standard deviation. Standard errors for equilibrium dissociation constants are listed in **Supplementary Tables S11 and S12**. Serotonin sensor sequences used were derived from Nakatsuka *et al*., 2018 as follows^3^ : *FAM-TAM labeled sensor: /5Biosg/AAA AAA AAA GAC TGG TAG GCA GAT AGG GGA AGC TGA T/i6-FAMK/T TCG ATG CGT GGG TC/36-TAMTSp/. BHQ-1-Cy3 sensor: /5Biosg/TTT TTT GAC TGG TAG GCA GAT AGG GGA AGC TGA T/iBHQ-1/TT CGA TGC GTG GGT C/3Cy3Sp/. Cy3-Cy5 sensor: AAA AAA AAA AAA AAA AAG ACT GGT AGG CAG ATA GGG GAA GCT GAT /iCy3/TTC GAT GCG TGG GTC /3Cy5Sp/. The serotonin DBS sequences used were as follows. Cy3 – BHQ-1 position 1: (aptamer strand): AAA TAC AAC AAG AAA AAA CTC TCG GGA CG/iCy3/ ACT GGT AGG CAG ATA GGG GAA GCT GAT TCG ATG CGT GGG TCG TCC C and (displacement strand) /5BHQ_1/CG TCC CGA GAG AAC ATT CTC ACC TTA CAC ATT TTA TTC CCA TTT TCT TGT TGT ATT T/3Bio/. Cy3 – BHQ-1 position 2: (aptamer strand) AAA TAC AAC AAG AAA AAA CTC TC/iCy3/ GGG ACG ACT GGT AGG CAG ATA GGG GAA GCT GAT TCG ATG CGT GGG TCG TCC C and (displacement strand) CGT CCC /iBHQ-1/GA GAG AAC ATT CTC ACC TTA CAC ATT TTA TTC CCA TTT TCT TGT TGT ATTT /3Bio/. Cy3 – BHQ-1 or BHQ-2 position 3: (aptamer strand) AAA ATA CAA CAA GAA AAA A/iCy3/C TCT CGG GAC GAC TGG TAG GCA GAT AGG GGA AGC TGA TTC GAT GCG TGG GTC GTC CC and (displacement strand) CGT CCC GAG AGX AAC ATT CTC ACC TTA CAC ATT TTA TTC CCA TTT TCT TGT TGT ATT T/3Bio/, where X is BHQ-1 or BHQ-2.*

**Supplementary Tables**

**Supplementary Table S1.** **DNA sequences of DBS, MAB, and controls used in this work**

Sequences used in the work are listed below. The **aptamer** sequence is in bold. The anchor section is underlined. The *linker* section is italicized. A nucleotide insertion is indicated by brackets. Note, modifications (X, Y, Z, X’) employed depend on the desired sequence; refer to **Supplementary Table S2**.

| **#** | **Target** | **Architecture** | **Strand** | **Bases** | **Sequence (5’ – 3’)** |
| --- | --- | --- | --- | --- | --- |
| 1 | Dopamine | DBS | Aptamer | 75/76^A^ | AAA TAC AAC AAG AAA AAA **CTC TC**X**G GGA**Y **CGA CG**Z(T)^A^**C CAG TTT GAA GGT TCG TTC GCA GGT GTG GAG TGA CGT CGT CCC**X’ |
| 2 | Dopamine | DBS | Displacement | 42/43^B^ | V(+T)CWG TCG XTCC CY*CT* *CTT CAC ATA TCT A*TT TTT TCT TGT TGT ATT TZ |
| 3 | Dopamine | MAB | Aptamer | 40 | X**ACG** **CCA GTT TGA AGG TTC GTT CGC AGG TGT GGA GTG ACG** **T**Y |

^A^i6-FAMK (Internal 6-FAM by azide) modification is commercially (by IDT) incorporated on a dT, whereas for Cy3 (+T) the dT is intentionally inserted (by researcher) at the bracketed position. ^B^In the terminal offset a dT is intentionally added at the bracketed position (5’ end). The 3Dab modification in the MAB is commercially incorporated on a dC.

**Supplementary Table S2**. **Legend describing the sequence modifications performed in this work.** Modification abbreviations follow IDT modification codes. Note, not all modification combinations were tested in this work; please refer to the combinations used. Example: The DBS Cy3–BHQ-1 sensor for position 1 was assembled using a (DBS) architecture, which requires two components. The aptamer strand was created from DBS aptamer (sequence 1) by replacing the placeholder 'Z' with an internal Cy3 (iCy3) modification. The corresponding displacement strand was created from DBS displacement (sequence 2), with a quencher (5BHQ_1) incorporated at position 'V'.

| **Architecture** | **Strand (Sequence #)** | **Description** | **Modification** | |  |
| --- | --- | --- | --- | --- | --- |
| **DBS** | **Aptamer (1)** | Position 1 | Z = iCy3, i6-FAMK, or iBHQ-1 | |  |
|  |  | Position 2 | Y = iCy3 | |  |
|  |  | Position 3 | X = iCy3 | |  |
|  |  | Position 1 + dT | Z = iCy3 and (+T) | |  |
|  |  | Cy3Cy5/No | X = iCy3  X’ = 3Cy5Sp | |  |
|  | **Displacement (2)** | Position 1 | V = 5BHQ_1, 5IABkFQ, or 5Cy3 |  |  |
|  |  | Position 2 | X = iBHQ-1  Z = 3Bio | |  |
|  |  | Position 3 | Y = iBHQ-1  Z = 3Bio | |  |
|  |  | Cy3Cy5/No | Z = 3Bio | |  |
|  |  | Position 1 Int. Offset | W = iBHQ-1  Z= 3Bio | |  |
|  |  | Position 1 Term. Offset | V = 5BHQ_1 and (+T)  Z = 3Bio | |  |
| **MAB** | **Aptamer (3)** | Standard | X = Cy3, 56-FAM, or 5BHQ_1  Y = 3BHQ_1, 3BHQ_2, 3Dab, or 36-FAM | |  |

**Supplementary Table S3. Apparent equilibrium dissociation constants for Cy3 – BHQ-1 MAB and FAM – BHQ-1 DBS over time**

| **Construct** | **Time (min)** | ***K*_d_ (µM)** |
| --- | --- | --- |
| Cy3 – BHQ-1 (MAB) | 5 | 57.6 ± 5.7 |
|  | 15 | 77.7 ± 6.6 |
|  | 30 | 44.0 ± 5.5 |
| FAM (+T) – BHQ-1 (DBS) | 5 | 6.1 ± 0.7 |
|  | 15 | 6.4 ± 0.8 |
|  | 30 | 6.3 ± 0.9 |

^*^Error is standard error of triplicate measurements.

**Supplementary Table S4. Apparent equilibrium dissociation and time constants for DBS with varying fluorescent reporter pair combinations**

| **Modification (Apt – Dis)** | ***K*_d_ (µM)** | ***τ* (s)** |
| --- | --- | --- |
| Cy3 – BHQ-1 | 229.9 ± 22.0 | 995.0 ± 8.1 |
| FAM (+T) – BHQ-1 | 6.1 ± 0.7 | 206.2 ± 0.8 |
| Cy3 (+T) – BHQ-1 | 76.7 ± 5.5 | 886.8 ± 1.9 |
| BHQ-1 – Cy3 (1:1) | 95.6 ± 6.7 | 344.5 ± 1.8 |
| Cy3 – IABkFQ | 85.0 ± 15.6 | 648.8 ± 4.2 |
| FAM (+T) – IABkFQ | 19.6 ± 0.9 | 601.6 ± 0.9 |
| Cy3 (+T) – IABkFQ | 41.8 ± 5.0 | 701.0 ± 2.1 |
| Cy3/Cy5 – No | 9.9 ± 3.6^A^ (5.4 ± 0.9)^B^ | 69.8 ± 2.8 |

^A^K_d_ calculated with 520 nm excitation and 565 nm emission. K_d_ calculated using the ratio of 670 nm and 565 nm emission with 520 nm. excitation. Error is standard error of triplicate measurements.

**Supplementary Table S5. Apparent equilibrium dissociation and time constants for DBS using Cy3** – **BHQ-1 with varying offsets**

| **Modification (Apt – Dis)** | **Offset** | ***K*_d_ (µM)** | ***τ* (s)** |
| --- | --- | --- | --- |
| Cy3 – BHQ-1 | Adjacent | 229.9 ± 22.0 | 995.0 ± 8.1 |
|  | Terminal | 142.9 ± 11.7 | 565.7 ± 3.2 |
|  | Internal | 34.0 ± 3.3 | 407.0 ± 1.1 |
| Cy3 (+T) – BHQ-1 | Adjacent | 76.7 ± 5.5 | 886.8 ± 1.9 |
|  | Terminal | 40.1 ± 5.0 | 572.9 ± 2.1 |
|  | Internal | 17.3 ± 1.3 | 304.0 ± 1.2 |
| FAM (+T) – BHQ-1 | Adjacent | 6.1 ± 0.7 | 206.2 ± 0.8 |
|  | Terminal | 10.2 ± 1.0 | 240.9 ± 0.7 |
|  | Internal | 7.5 ± 0.9 | 196.6 ± 0.9 |

Error is standard error of triplicate measurements.

**Supplementary Table S6. Apparent equilibrium dissociation and time constants for DBS using Cy3** – **BHQ-1 varying aptamer:displacement ratios**

| **Modification (Apt – Dis)** | **Ratio (Apt:Dis)** | ***K*_d_ (µM)** | ***τ* (s)** |
| --- | --- | --- | --- |
| Cy3 – BHQ-1 | 1:4 | 229.9 ± 22.0 | 995.0 ± 8.1 |
|  | 1:1 | 175.6 ± 13.3 | 601.6 ± 0.9 |
| BHQ-1 – Cy3 | 4:1 | 107.2 ± 6.0 | 314.5 ± 0.7 |
|  | 1:1 | 95.6 ± 6.7 | 344.5 ± 1.8 |

Error is standard error of triplicate measurements.

**Supplementary Table S7.** **Apparent equilibrium dissociation and time constants using Cy3 – BHQ-1 with varying positions**

| **Position** | ***K*_d_ (µM)** | ***τ* (s)** |
| --- | --- | --- |
| 1 | 229.9 ± 22.0 | 995.0 ± 8.1 |
| 2 | 2.2 ± 0.7 | N/A |
| 3 | 2.3 ± 0.3 | 12.0 ± 0.4 |
| 3 (Cy3/Cy5) | 9.9 ± 3.6 | 69.8 ± 2.8 |

*Temporal response for Position could not be confidently fit due to the limitation of the instrument. Error is standard error of triplicate measurements.

**Supplementary Table S8. Sensor metrics for modification combinations and different sensor architectures**

| **Arch.** | **Construct** | ***K*_d_ (μM)** | **Max Signal (RFU)** | **Min Signal (RFU)** | **μ_Min_** | **[μ_Min_] (μM)** | **LOD**  **(μM)** | **Operating Range (μM) [Fold change]** | **Hill Coeff.** |
| --- | --- | --- | --- | --- | --- | --- | --- | --- | --- |
| DBS (Apt/Dis) | Cy3/BHQ-1 (1:4) | 229.9 | 30471.7 | 9411.6 | 1.51 | 79.7 | 9.73 | 9.2 - 622.3 | 1.10 |
|  | Cy3/BHQ-1 (1:1) | 175.6 | 25039.0 | 9316.9 | 1.38 | 70.8 | 2.96 | 5.7 – 758.6 | 1.05 |
|  | BHQ-1/Cy3 (4:1) | 107.2 | 21730.5 | 1543.8 | 1.28 | 54.9 | 2.02 | 3.3 – 707.9 | 1.14 |
|  | BHQ-1/Cy3 (1:1) | 96.7 | 25518.9 | 3620.0 | 1.42 | 49.6 | 0.61 | 4.4 – 452.9 | 1.16 |
|  | Cy3/IABkFQ | 85.0 | 27781.7 | 9498.7 | 2.17 | 40.4 | 0.74 | N/A | 1.32 |
|  | Cy3/BHQ-1 (int.) | 33.1 | 28047.7 | 9302.9 | 1.65 | 19.4 | 2.88 | 3.3 – 95.1 | 1.14 |
|  | Cy3/BHQ-1 (term.) | 142.9 | 23673.9 | 7670.8 | 1.42 | 62.8 | 6.20 | 5.6 – 591.6 | 1.05 |
|  | Cy3/BHQ-1 (P2) | 2.2 | 21048.7 | 16329.7 | 2.26 | 2.1 | 0.18 | N/A | 0.50 |
|  | Cy3/BHQ-1 (P3) | 2.3 | 20367.0 | 14781.5 | 2.15 | 2.2 | 0.73 | N/A | 0.89 |
|  | Cy3(+T)/BHQ-1 | 77.7 | 31749.5 | 5553.6 | 1.43 | 42.9 | 0.99 | 3.9 – 371.5 | 1.16 |
|  | Cy3(+T)/IABkFQ | 42.0 | 32569.3 | 9513.1 | 1.86 | 21.7 | 0.31 | 7.4 – 58.1 | 1.14 |
|  | Cy3(+T)/BHQ-1 (int.) | 17.6 | 33251.2 | 12234.8 | 1.50 | 13.1 | 1.12 | 1.5 – 87.9 | 1.07 |
|  | Cy3(+T)/BHQ-1 (term.) | 40.1 | 27927.5 | 6642.6 | 1.69 | 25.7 | 1.34 | 4.9 – 114.8 | 1.25 |
|  | FAM(+T)/BHQ-1 | 6.1 | 47527.3 | 20617.7 | 1.63 | 5.2 | 0.52 | 0.9 – 25.6 | 0.92 |
|  | FAM(+T)/IABkFQ | 20.0 | 45019.8 | 12755.9 | 1.27 | 12.7 | 0.48 | 0.7 – 168.3 | 0.94 |
|  | FAM(+T)/BHQ-1 (int.) | 7.0 | 42438.8 | 14775.6 | 1.83 | 5.4 | 0.09 | 1.8 – 15.3 | 1.13 |
|  | FAM(+T)/BHQ-1 (term.) | 10.3 | 43506.1 | 15541.0 | 1.68 | 7.4 | 0.28 | 1.5 – 31.3 | 0.92 |
|  | Cy3Cy5/No | 2.7 | 5696.8 | 3214.2 | 1.92 | 2.5 | 0.18 | 1.2 – 5.1 | 0.73 |
| MAB (5’/3’) | Cy3/BHQ-1 | 53.8 | 7929.2 | 3808.8 | 2.01 | 24.9 | 11.32 | N/A | 0.79 |
|  | Cy3/BHQ-2 | 33.6 | 5446.0 | 3264.9 | 2.63 | 13.1 | 22.71 | N/A | 0.83 |
|  | Cy3/Dab | 199.8 | 6734.2 | 1364.4 | 3.42 | 52.7 | 29.09 | N/A | 0.75 |
|  | FAM/BHQ-1 | 3.0 | 8965.6 | 2060.4 | 1.23 | 3.6 | 0.03 | 0.2 – 47.0 | 0.91 |
|  | BHQ-1/FAM | 5.3 | 7379.6 | 2348.5 | 1.90 | 4.3 | 0.53 | 1.9- 9.5 | 1.26 |
|  | FAM/Dab | 3.4 | 11846.5 | 5432.7 | 1.36 | 3.7 | 1.33 | 0.3 – 32.4 | 0.98 |

*The resolution of molecular concentration (μMin), limit of detection (LOD), operating range, and sensitivity (Hill coefficient) were calculated as described in **Supplementary Note 3**. Data normalization and fits are detailed in the **Materials and Methods Section** and in **Supplementary Note 1**. Standard errors for I_d_, max signal, and min signal available in Source Data File.

**Supplementary Table S9.** **Apparent equilibrium dissociation and time constants for MAB with varying fluorescent reporter pair combinations**

| **Modification (5’ – 3’)** | ***K*_d_ (µM)** | ***τ* (s)** |
| --- | --- | --- |
| Cy3 – BHQ-1 | 53.8 ± 8.8 | N/A |
| FAM – BHQ-1 | 3.0 ± 0.1 | N/A |
| Cy3-Dab | 199.8 ± 88.5^A^ | N/A |
| FAM-Dab | 3.2 ± 0.2 | N/A |
| BHQ-1-FAM | 5.3 ± 0.8 | N/A |
| Cy3-BHQ-2 | 12.4 ± 2.0 | N/A |

*Temporal response for the MAB were faster than could be observed due to the minimum detection time the instrument. **^A^***K*_d_ is likely overestimated, and the standard error is extremely high because the trace does not plateau, presumably due to polydopamine formation (**Supplementary Figure S3**). Error is standard error of triplicate measurements.

**Supplementary Table S10.** **Apparent equilibrium dissociation constants for ATP-ISD with varying fluorescent reporter pair combinations**

| **Construct** | ***K*_d_ (µM)** |
| --- | --- |
| Cy3 – BHQ-1 | 460 ± 65 |
| Cy3 – Dab | 390 ± 83 |
| FAM – BHQ-1 | 1800 ± 1700 |
| FAM – Dab | 1300 ± 600 |

Error is standard error of triplicate measurements.

**Supplementary Table S11.** **Apparent equilibrium dissociation constants for dual-labeled serotonin sensor with varying fluorescent reporter pair combinations**

| **Construct** | ***K*_d_ (nM)** |
| --- | --- |
| FAM – TAM | 120 ± 18 |
| BHQ-1 – Cy3 | 100 ± 59 |
| Cy3 – Cy5 | 890 ± 470 |

Error is standard error of triplicate measurements.

**Supplementary Table S12.** **Apparent equilibrium dissociation constants for serotonin DBS with varying fluorescent reporter pair positions**

| **Construct** | ***K*_d_ (nM)** |
| --- | --- |
| Cy3 – BHQ-1 Position 1 | N/A |
| Cy3 – BHQ-1 Position 2 | N/A |
| Cy3 – BHQ-1 Position 3 | 110 ± 18 |
| Cy3 – BHQ-2 Position 3 | 110 ± 36 |

Error is standard error of triplicate measurements. Cy3 – BHQ-1 at positions 1 and 2 could not be fit according to the method detailed in **Supplementary Note 1**.

**Supplementary Table S13. Apparent melting temperatures for constants DBS and MAB with varying fluorescent reporter pair combinations and positions**

| **Architecture** | **Construct** | **T_m_ in the absence of dopamine (°C)** | **T_m_ in the presence of 50 μM dopamine (°C)** |
| --- | --- | --- | --- |
| DBS (Apt/Dis) | Cy3/BHQ-1 (1:4) | 51.7 ± 0.1 | 50.9 ± 0.1 |
|  | Cy3/BHQ-1 (1:1) | 49.3 ± 0.1 | 47.7 ± 0.2 |
|  | BHQ-1/Cy3 (4:1) | 54.1 ± 0.1 | 53.8 ± 0 |
|  | BHQ-1/Cy3 (1:1) | 51.0 ± 0 | 50.6 ± 0.2 |
|  | Cy3/IABkFQ | 48.6.± 0 | N/A |
|  | Cy3/BHQ-1 (int.) | 50.9 ± 0.1 | 50.4 ± 0 |
|  | Cy3/BHQ-1 (term.) | 51.2 ± 0 | 50.6 ± 0 |
|  | Cy3/BHQ-1 (P3) | 50.3 ± 0.1 | 50.1 ± 0.1 |
|  | Cy3(+T)/BHQ-1 | 51.6 ± 0 | 50.9 ± 0.1 |
|  | Cy3(+T)/BHQ-1 (int.) | 50.3 ± 0.1 | 49.9 ± 0.1 |
|  | Cy3(+T)/BHQ-1 (term.) | 50.8 ± 0 | 50.0 ± 0.1 |
|  | FAM(+T)/BHQ-1 | 49.1 ± 0 | 48.9 ± 0.1 |
|  | FAM(+T)/IABkFQ | 47.3.± 0.2 | N/A |
|  | FAM(+T)/BHQ-1 (int.) | 49.1 ± 0.1 | 49.3 ± 0.1 |
|  | FAM(+T)/BHQ-1 (term.) | 48.4 ± 0 | 48.3 ± 0.1 |
| MAB (5’/3’) | Cy3/BHQ-1 | 49.5 ± 0.3 | 52.7 ± 0.8 |
|  | Cy3/BHQ-2 | 49.4 ± 0.8 | 53.3 ± 0.8 |
|  | Cy3/Dab | 43.3 ± 1.0 | 53.6 ± 0.3 |
|  | FAM/BHQ-1 | 44.8 ± 0 | 37.7 ± 0.1 |
|  | BHQ-1/FAM | 40.0 ± 0.2 | 37.3 ± 0.1 |
|  | FAM/Dab | 41.7 ± 0.2 | 36.9 ± 0.1 |

*The melting temperatures were calculated as described in the **Materials and Methods Section**. Error represents the standard deviation of triplicate measurements.

**Supplementary Notes**

**Supplementary Note 1**

**Method of analysis for apparent binding affinity and observed kinetics of aptamer-switch sensors**

The dopamine aptamer was assumed to follow one-site binding. Raw fluorescence data were first background corrected, then normalized to the highest value. All normalized binding curves were fit to the Hill1 equation in Origin, where n = 1, x is the dopamine concentration, and k is the apparent equilibrium dissociation constant (*K*_d_):

$$y=START+\left( END-START \right)\frac{x}{k+x} (1)$$

The dopamine aptamer was assumed to follow single-phase kinetics. Raw fluorescence data were first background corrected, and normalized to the highest value. All normalized observed kinetic traces were fit to the following equation, where y_0_ is the offset, A is the amplitude, x is the dopamine concentration, and t is the observed time constant (***τ*)**:

$$y=y_{0}+A\left( 1-e^{-\frac{x}{t}} \right) (2)$$

**Supplementary Note 2**

**Estimating degrees of freedom/motion: Angle range at which FRET is prevalent in DBS system at both terminal and internal positions (positions 1, 2 and 3):**

To define the performance boundaries of the system, we performed calculations for two opposing scenarios: a fully collapsed state, representing the minimum dye-quencher separation (the "worst-case" for signal), and a fully stretched state, representing the maximum separation. We set the internucleotide distance for single-stranded DNA= 0.34 nm. For each modification position, we will calculate the conformational space—the range of possible angles—that places the fluorophore and quencher within the effective Förster distance for FRET (typically under 10 nm). We hypothesize that a larger accessible space for FRET in the unbound state will result in signal quenching, which in turn will reduce the sensor's overall dynamic range.

**For positions 2 and 3:**

For a system with sides A = 1.7 (nm) and B = 5.1 (nm), we want to find the range of the angle γ (between sides A and B) for which the third side C is less than 5 (nm).

We use the Law of Cosines: C^2^=A^2^+B^2^−2ABcos(γ).

We want C<5, so C^2^<52=25 nm^2^.

Thus, A^2^+B^2^−2ABcos(γ)<25.

Let's plug in the values for A and B:

- A=1.7⟹A^2^=2.89 nm^2^
- B=5.1⟹B^2^=26.01 nm^2^
- 2AB=2×1.7×5.1=17.34 nm^2^

The inequality becomes: 2.89+26.01−17.34cos(γ)<25

γ<77.00 (approx) The angle γ must be in the range: 0 ≤γ<77.00.

**For positions 1 (optimal):**

For between sides A=4.7 nm and B=8.5nm for which the third side C is less than 5 nm.

We use the Law of Cosines: C^2^=A^2^+B^2^−2ABcos(γ).

We want to find γ such that C<5 nm, which means C^2^<52=25 nm^2^.

So, A^2^+B^2^−2ABcos(γ)<25.

Plugging in the values for A and B:

- A=4.7 nm⟹A^2^=22.09 nm^2^
- B=8.5 nm⟹B^2^=72.25 nm^2^
- 2AB=2×4.7×8.5=79.9 nm^2^

The inequality becomes: 22.09+72.25−79.9cos(γ)<25;

The angle γ must be in the range: 0≤γ<29.79.

**Supplementary Note 3**

**Comparison of sensor metrics – Resolution of molecular concentration, limit of detection, operating range, and sensitivity**

For the various fluorescent modification combinations, we compared the resolution of molecular concentration (RMC), the limit of detection (LOD), operating range, and sensitivity. The RMC was computed using the code provided by Wilson *et al*.^6^, for a two-tailed t-test where α is 0.01 with four degrees of freedom (critical value = 4.604) and for a 95% confidence interval (Z = 1.960) unless otherwise specified. The LOD was calculated as the concentration that correlated to the signal of the sum of three standard deviations of the background and the background itself. The operating range was arbitrarily defined as the range of concentrations of RMC values less than two. Sensitivity was calculated by fitting binding curve data to the Hill1 equation in Origin.

Generally, the concentration of μ_Min_ and LOD follows trends in *K*_d_ as expected, because they are inherently coupled. There do not appear to be any significant differences or trends concerning these metrics for the DBS concerning the modification combination (**Supplementary Figure S12 and Supplementary Table S8**). However, we mention that for the MAB, the Cy3 combinations (BHQ-1, BHQ-2, Dab) were measured with lowered excitation wavelength and significantly greater gain (**Materials and Methods Section**). Consequently, their true resolution (sensor metrics) is significantly worse than what we illustrate (**Figure 5**). This potentially indicates that the MAB, as an off-switch, is significantly more prone to negative structural impacts of these Cy3 combination modifications (i.e. the Cy3 destabilizes the shorter MAB proposed aptamer stem more than the longer stem present in the DBS aptamer, thus diminishing the effective affinity of the aptamer, as stem formation has been shown to be important target-binding),^7^ compared to the DBS an on-switch. As a result, the DBS and strand displacement architectures could be more robust to these dye modifications.

In terms of operating range, while the maximal fold change in raw signal is on average around 3 for all constructs, the fold resolvable operating range varied significantly (4.2 for Cy3Cy5 DBS to 310.4 for FAM **–** BHQ-1 MAB), and often did not correlate with each other. On average, Cy3-BHQ-1 had the best fold operating range at around 130, nevertheless consistent with their poor affinity, it had the highest lower bound of their operating range, averaging 5.6 μM. The FAM combinations had the best operating range lower bound of 0.8 μM, however, while the FAM **–**IABkFQ combination had the greatest fold operating range of the DBS at 243, FAM **–** BHQ-1 was 8-fold lower at 30, demonstrating most likely the photophysical impact of fluorophore-quencher selection (raw fluorescence minimum of FAM **–** BHQ-1 and IABkFQ was 13000 and 21000, respectively, though this logic does not apply to all cases, indicating a more complex explanation). Regarding the placement of Cy3 **–** BHQ-1 at positions 2 and 3, we speculate that the reduction of dye flexibility significantly decreases the quenching efficiency and maximal signal change, leading to no RMC values under 2 for all concentrations. Thus, gains in affinity and kinetics at this position are balanced by a loss of resolution. Comparing the DBS to MAB, presumably due to the difference in their architectures and structural interactions (mentioned above), the Cy3 combinations in a MAB possess very poor resolution, with no concentrations with RMC values under two, while previously they had the largest fold resolvable range for the DBS. On the other hand, the fold resolvable operating range improved 10 times for FAM-BHQ-1 from 30 to 310 for the DBS to the MAB, respectively.

**References**

(1) Moreira, B. G.; You, Y.; Behlke, M. A.; Owczarzy, R. Effects of Fluorescent Dyes, Quenchers, and Dangling Ends on DNA Duplex Stability. *Biochem. Biophys. Res. Commun.* **2005**, *327* (2), 473–484. https://doi.org/10.1016/j.bbrc.2004.12.035.

(2) Hemmatpour, H.; De Luca, O.; Crestani, D.; Stuart, M. C. A.; Lasorsa, A.; Van Der Wel, P. C. A.; Loos, K.; Giousis, T.; Haddadi-Asl, V.; Rudolf, P. New Insights in Polydopamine Formation via Surface Adsorption. *Nat. Commun.* **2023**, *14* (1). https://doi.org/10.1038/s41467-023-36303-8.

(3) Nakatsuka, N.; Yang, K.-A.; Abendroth, J. M.; Cheung, K. M.; Xu, X.; Yang, H.; Zhao, C.; Zhu, B.; Rim, Y. S.; Yang, Y.; Weiss, P. S.; Stojanović, M. N.; Andrews, A. M. Aptamer–Field-Effect Transistors Overcome Debye Length Limitations for Small-Molecule Sensing. *Science* **2018**, *362* (6412), 319–324. https://doi.org/10.1126/science.aao6750.

(4) Hariri, A. A.; Cartwright, A. P.; Dory, C.; Gidi, Y.; Yee, S.; Thompson, I. A. P.; Fu, K. X.; Yang, K.; Wu, D.; Maganzini, N.; Feagin, T.; Young, B. E.; Afshar, B. H.; Eisenstein, M.; Digonnet, M. J. F.; Vuckovic, J.; Soh, H. T. Modular Aptamer Switches for the Continuous Optical Detection of Small‐Molecule Analytes in Complex Media. *Adv. Mater.* **2024**, *36* (1), 2304410. https://doi.org/10.1002/adma.202304410.

(5) Wilson, B. D.; Hariri, A. A.; Thompson, I. A. P.; Eisenstein, M.; Soh, H. T. Independent Control of the Thermodynamic and Kinetic Properties of Aptamer Switches. *Nat. Commun.* **2019**, *10* (1), 5079. https://doi.org/10.1038/s41467-019-13137-x.

(6) Wilson, B. D.; Eisenstein, M.; Soh, H. T. Comparing Assays via the Resolution of Molecular Concentration. *Nat. Biomed. Eng.* **2021**, *6* (3), 227–231. https://doi.org/10.1038/s41551-021-00832-8.

(7) Liu, X.; Hou, Y.; Chen, S.; Liu, J. Controlling Dopamine Binding by the New Aptamer for a FRET-Based Biosensor. *Biosens. Bioelectron.* **2021**, *173*, 112798. https://doi.org/10.1016/j.bios.2020.112798.
